# Supplementary material for: Protein sequences bound to mineral surfaces persist into deep time
Source: eLife. 2016 Sep 27;5:e17092. doi: 10.7554/eLife.17092 (PMC5039028; doi:10.7554/eLife.17092)
Supplement: Supplementary file 1. — The proteins identified in each ostrich eggshell sample are reported, together with the number of identified peptides and the percentage coverage, Val D/L value and hydropathicity. DOI: http://dx.doi.org/10.7554/eLife.17092.020 [file elife-17092-supp1.docx]

| **Sample ID** | | **Modern OES** | **Elands Bay Cave_LOT 1872** | **Elands Bay Cave_LOT 1866** | **Elands Bay Cave_LOT 1849** | **Elands Bay Cave_LOT 1823** | **Elands Bay Cave_LOT 1868** | **Elands Bay Cave_LOT 1850** | **Elands Bay Cave_LOT 1840** | **Elands Bay Cave_LOT 1819** | **Pinnacle Point 5-6_LOT 4613** | **Pinnacle Point 5-6_LOT 4652** | **Pinnacle Point 5-6_LOT 4671** | **Pinnacle Point 5-6_LOT 4649** | **Pinnacle Point 5-6_LOT 4675** | **Pinnacle Point 30_LOT 4697** | **Pinnacle Point 30_LOT 4683** | **Pinnacle Point 5-6_LOT 4605** | **Wonderwerk_LOT 14426** | **Olduvai_LOT 15575 T** | **Olduvai_LOT 15578 T** | **Olduvai_LOT 15579 E** | **Olduvai_LOT 15582 E** | **Laetoli_13902 E** | **Laetoli_13902 T** | **Laetoli_13898 E** | **Laetoli_13898 T** | **Laetoli_13901 E** | **Laetoli_13901 N** | **Laetoli_13901 T** | **Total** |
| --- | --- | --- | --- | --- | --- | --- | --- | --- | --- | --- | --- | --- | --- | --- | --- | --- | --- | --- | --- | --- | --- | --- | --- | --- | --- | --- | --- | --- | --- | --- | --- |
| Hydroapathicity | | -6.5 | -6.4 | -4.8 | -5.8 | -5.7 | -5.7 | -5.5 | -5.8 | -5.7 | -10.2 | -5 | -5.4 | -7 | -5.9 | -6.1 | -5.3 | -6.3 | -5.9 | 24.4 | 11.8 | 17.3 | -17.2 | -17.2 | -10.8 | -15.9 | -16.4 | -12.9 | -17.1 | -16.6 |  |
|  |  |  |  |  |  |  |  |  |  |  |  |  |  |  |  |  |  |  |  |  |  |  |  |  |  |  |  |  |  |  |  |
| **Val D/L** | | 0.001 | 0.025 | 0.028 | 0.053 | 0.073 | 0.075 | 0.08 | 0.095 | 0.112 | 0.21 | 0.233 | 0.242 | 0.255 | 0.279 | 0.368 | 0.373 | 0.525 | 0.855 | 1.005 | 0.999 | 1.007 | 1.012 | 1.12 | 1.12 | 1.17 | 1.17 | 1.16 | 1.16 | 1.16 |  |
| **RecName: Full=Struthiocalcin-1; Short=SCA-1** | gi\|46396750 | 346 | 534 | 445 | 453 | 410 | 509 | 340 | 399 | 282 | 60 | 262 | 289 | 231 | 334 | 282 | 315 | 218 | 13 | 1 | 3 | 5 | 4 | 8 | 7 | 3 | 2 | 9 | 7 | 5 | 5776 |
| **RecName: Full=Struthiocalcin-2; Short=SCA-2** | gi\|46396751 | 222 | 363 | 313 | 269 | 205 | 368 | 186 | 194 | 110 | 17 | 116 | 134 | 86 | 162 | 119 | 145 | 86 |  |  |  |  |  |  |  |  |  |  |  |  | 3095 |
| **von Willebrand factor** | gi\|697484581 | 382 | 200 | 243 | 178 | 162 | 256 | 152 | 202 | 52 | 0 | 65 | 66 | 21 | 102 | 68 | 115 | 45 |  |  |  |  |  |  |  |  |  |  |  |  | 2309 |
| **von Willebrand factor partial** | gi\|678214590 | 379 |  |  |  |  |  |  |  | 52 | 0 |  | 66 | 21 |  | 68 | 115 |  |  |  |  |  |  |  |  |  |  |  |  |  | 701 |
| **Aggrecan core protein** | gi\|697501075 | 209 | 150 | 148 | 117 | 76 | 154 | 106 | 100 | 50 | 5 | 60 | 47 | 21 | 93 | 62 | 82 | 44 |  |  |  |  |  |  |  |  |  |  |  |  | 1524 |
|  | gi\|678217626 | 209 | 150 | 148 | 117 | 76 | 154 | 106 | 100 | 50 | 5 | 60 |  | 21 | 93 | 62 | 82 | 44 |  |  |  |  |  |  |  |  |  |  |  |  | 1477 |
| **iron binding protein** | gi\|50892957 | 242 | 228 | 164 | 58 | 58 | 169 | 84 | 65 | 24 |  | 39 | 19 | 13 | 30 | 32 | 43 | 11 |  |  |  |  |  |  |  |  |  |  |  |  | 1279 |
| **vitelline membrane outer layer protein 1-like** | gi\|697508924 | 126 | 71 | 66 | 47 | 35 | 84 | 33 | 34 | 21 |  | 18 | 12 | 5 | 17 | 8 | 22 | 5 |  |  |  |  |  |  |  |  |  |  |  |  | 604 |
| **Apolipoprotein D** | gi\|678221588 | 77 | 95 | 77 | 55 | 34 | 35 | 39 | 42 | 28 |  | 11 | 11 | 9 | 19 | 17 | 12 | 1 |  |  |  |  |  |  |  |  |  |  |  |  | 562 |
|  | gi\|697523391 | 77 | 95 | 77 | 55 | 34 | 35 | 39 | 42 | 28 |  | 11 | 11 | 9 | 19 | 17 | 12 | 1 |  |  |  |  |  |  |  |  |  |  |  |  | 562 |
| **tenascin isoform X3** | gi\|697455783 | 89 | 33 | 45 | 29 | 17 | 74 | 32 | 36 | 7 |  | 3 | 17 | 1 | 19 | 12 | 20 | 3 |  |  |  |  |  |  |  |  |  |  |  |  | 437 |
| **serotransferrin** | gi\|697445159 | 242 |  |  | 56 | 56 |  | 80 |  |  |  |  |  |  |  |  |  |  |  |  |  |  |  |  |  |  |  |  |  |  | 434 |
| **BPI fold-containing family B member 4 partial** | gi\|697430975 | 33 | 86 | 96 | 38 | 42 | 59 | 28 | 52 | 24 | 2 | 27 | 28 | 11 | 22 | 26 | 29 | 13 |  |  |  |  |  |  |  |  |  |  |  |  | 616 |
|  | gi\|678205748 | 26 | 43 | 51 | 29 | 17 | 47 | 20 | 20 | 11 |  | 16 | 15 | 0 | 13 | 16 | 14 | 6 |  |  |  |  |  |  |  |  |  |  |  |  | 344 |
|  | gi\|697430934 | 26 | 43 | 51 | 29 | 17 | 47 | 20 | 20 | 11 |  | 16 | 15 | 0 | 13 | 16 | 14 | 6 |  |  |  |  |  |  |  |  |  |  |  |  | 344 |
|  | gi\|678205751 |  |  |  |  | 42 |  | 28 |  | 24 | 2 | 27 | 28 |  | 22 | 26 | 29 | 13 |  |  |  |  |  |  |  |  |  |  |  |  | 241 |
| **tenascin isoform X1** | gi\|697455750 |  | 33 | 45 | 29 | 17 | 74 | 32 | 36 | 7 |  | 3 |  | 1 | 19 | 12 | 20 | 3 |  |  |  |  |  |  |  |  |  |  |  |  | 331 |
| **tenascin isoform X2** | gi\|697455757 |  | 33 |  | 29 | 17 |  | 32 | 36 | 7 |  |  |  | 1 |  | 12 |  | 3 |  |  |  |  |  |  |  |  |  |  |  |  | 170 |
| **mucin-5AC** | gi\|697481828 | 82 | 14 | 27 | 22 | 28 | 32 | 24 | 15 | 9 |  | 9 | 10 | 1 | 18 | 14 | 11 | 7 |  |  |  |  |  |  |  |  |  |  |  |  | 323 |
| **carnitine O-palmitoyltransferase 1 muscle isoform** | gi\|697511083 |  |  |  | 97 |  |  |  |  |  |  | 41 |  |  | 46 | 42 | 48 |  |  |  |  |  |  |  |  |  |  |  |  |  | 274 |
| **serum albumin-like** | gi\|697509029 | 114 | 24 | 30 | 6 | 13 | 30 | 3 | 8 | 2 |  | 3 | 1 |  | 1 | 2 | 5 |  |  |  |  |  |  |  |  |  |  |  |  |  | 242 |
| **Serum albumin partial** | gi\|678218948 |  | 24 | 30 | 6 | 13 | 30 |  | 8 | 2 |  | 3 | 1 |  | 1 | 2 | 5 |  |  |  |  |  |  |  |  |  |  |  |  |  | 125 |
| **immunoglobulin M heavy chain constant region transmemebrane form partial** | gi\|375162646 |  |  | 14 | 8 | 28 | 33 | 8 | 8 | 3 |  | 10 | 3 | 5 | 8 | 6 | 16 |  |  |  |  |  |  |  |  |  |  |  |  |  | 150 |
| **immunoglobulin M heavy chain constant region secretory form partial** | gi\|375162644 | 42 | 17 | 14 | 8 | 28 | 33 | 8 | 8 | 3 |  | 10 | 3 | 5 | 8 | 6 | 16 |  |  |  |  |  |  |  |  |  |  |  |  |  | 209 |
| **immunoglobulin A heavy chain constant region secretory form partial** | gi\|375162648 | 76 | 21 | 17 |  | 13 | 18 | 4 | 15 | 1 |  | 2 | 1 |  | 5 | 2 |  |  |  |  |  |  |  |  |  |  |  |  |  |  | 175 |
| **immunoglobulin lambda constant region partial** | gi\|375162654 | 24 | 16 | 9 | 6 | 14 | 19 | 2 | 13 |  |  | 1 |  |  | 4 | 2 | 3 |  |  |  |  |  |  |  |  |  |  |  |  |  | 113 |
| **mesothelin isoform X1** | gi\|697470177 | 63 | 16 | 28 | 9 | 6 | 35 | 2 | 3 | 3 |  | 2 |  |  |  |  | 2 |  |  |  |  |  |  |  |  |  |  |  |  |  | 169 |
|  | gi\|697470179 | 63 | 16 | 28 | 9 | 6 | 35 | 2 | 3 | 3 |  | 2 |  |  |  |  | 2 |  |  |  |  |  |  |  |  |  |  |  |  |  | 169 |
| **mesothelin isoform X2** | gi\|697470183 |  | 16 | 28 | 9 | 6 |  | 2 | 3 | 3 |  | 2 |  |  |  |  | 2 |  |  |  |  |  |  |  |  |  |  |  |  |  | 71 |
| **mesothelin isoform X3** | gi\|697470185 |  | 16 | 28 | 9 | 6 |  | 2 | 3 | 3 |  | 2 |  |  |  |  | 2 |  |  |  |  |  |  |  |  |  |  |  |  |  | 71 |
| **mesothelin isoform X4** | gi\|697470189 |  | 16 | 28 | 9 | 6 |  | 2 | 3 | 3 |  | 2 |  |  |  |  | 2 |  |  |  |  |  |  |  |  |  |  |  |  |  | 71 |
| **carbonic anhydrase 4** | gi\|697477202 | 84 | 21 | 10 | 4 | 4 | 17 | 2 | 4 | 2 |  |  |  |  | 2 |  |  |  |  |  |  |  |  |  |  |  |  |  |  |  | 150 |
| **Delta and Notch-like epidermal growth factor-related receptor partial** | gi\|678214778 | 38 | 12 | 8 | 6 | 7 | 13 | 4 | 5 | 1 |  |  | 1 |  |  | 1 | 1 |  |  |  |  |  |  |  |  |  |  |  |  |  | 97 |
| **uncharacterized protein LOC104140623** | gi\|697430936 | 32 | 6 | 35 |  |  | 15 | 4 |  | 2 |  |  |  |  |  |  |  |  |  |  |  |  |  |  |  |  |  |  |  |  | 94 |
| **Stanniocalcin-1 partial** | gi\|678209093 | 41 | 7 | 7 |  | 4 | 12 | 4 | 7 |  |  | 2 |  |  |  | 2 | 1 |  |  |  |  |  |  |  |  |  |  |  |  |  | 87 |
|  | gi\|697441180 | 41 | 7 | 7 |  | 4 | 12 | 4 | 7 |  |  | 2 |  |  |  | 2 | 1 |  |  |  |  |  |  |  |  |  |  |  |  |  | 87 |
| **polymeric immunoglobulin receptor** | gi\|697475274 | 37 | 5 | 4 | 4 | 8 | 13 | 4 | 6 |  |  | 3 |  |  |  |  | 1 |  |  |  |  |  |  |  |  |  |  |  |  |  | 85 |
|  | gi\|697475278 | 37 | 5 | 4 | 4 | 8 | 13 | 4 | 6 |  |  | 3 |  |  |  |  | 1 |  |  |  |  |  |  |  |  |  |  |  |  |  | 85 |
|  | gi\|697475280 | 37 | 5 | 4 | 4 | 8 | 13 | 4 | 6 |  |  | 3 |  |  |  |  | 1 |  |  |  |  |  |  |  |  |  |  |  |  |  | 85 |
| **Ovomucoid** | gi\|678219803 | 39 | 4 | 11 | 2 | 2 | 8 |  | 2 |  |  |  |  |  |  |  |  |  |  |  |  |  |  |  |  |  |  |  |  |  | 68 |
|  | gi\|697514088 | 39 | 4 | 11 | 2 | 2 | 8 |  | 2 |  |  |  |  |  |  |  |  |  |  |  |  |  |  |  |  |  |  |  |  |  | 68 |
| **beta-2-microglobulin** | gi\|697446419 | 22 | 7 | 7 | 4 | 4 | 11 | 4 | 3 | 2 |  | 1 | 1 |  | 1 |  | 1 |  |  |  |  |  |  |  |  |  |  |  |  |  | 68 |
| **Carbonic anhydrase 4 partial** | gi\|678213177 |  | 21 | 10 | 4 | 4 | 17 | 2 | 4 | 2 |  |  |  |  | 2 |  |  |  |  |  |  |  |  |  |  |  |  |  |  |  | 66 |
| **Pigment epithelium-derived factor** | gi\|678214846 | 28 | 6 | 7 | 5 |  | 3 | 1 | 5 | 2 |  |  | 2 |  | 2 |  | 1 |  |  |  |  |  |  |  |  |  |  |  |  |  | 62 |
|  | gi\|697485873 | 28 | 6 | 7 | 5 |  | 3 | 1 | 5 | 2 |  |  | 2 |  | 2 |  | 1 |  |  |  |  |  |  |  |  |  |  |  |  |  | 62 |
| **pantetheinase-like isoform X1** | gi\|697522911 | 28 | 6 | 5 | 6 | 3 | 5 | 2 | 2 |  |  |  |  |  | 1 |  |  |  |  |  |  |  |  |  |  |  |  |  |  |  | 58 |
| **pantetheinase-like isoform X2** | gi\|697522913 | 28 | 6 | 5 | 6 | 3 | 5 | 2 | 2 |  |  |  |  |  | 1 |  |  |  |  |  |  |  |  |  |  |  |  |  |  |  | 58 |
| **pantetheinase-like isoform X2** | gi\|697522916 | 28 | 6 | 5 | 6 | 3 | 5 | 2 | 2 |  |  |  |  |  | 1 |  |  |  |  |  |  |  |  |  |  |  |  |  |  |  | 58 |
| **Pantetheinase partial** | gi\|678221486 |  | 6 | 5 | 6 | 3 | 5 | 2 | 2 |  |  |  |  |  | 1 |  |  |  |  |  |  |  |  |  |  |  |  |  |  |  | 30 |
| **Golgi apparatus protein 1 partial** | gi\|678206587 | 45 |  | 7 |  |  | 5 |  |  |  |  |  |  |  |  |  |  |  |  |  |  |  |  |  |  |  |  |  |  |  | 57 |
| **Golgi apparatus protein 1** | gi\|697433909 | 45 |  |  |  |  | 5 |  |  |  |  |  |  |  |  |  |  |  |  |  |  |  |  |  |  |  |  |  |  |  | 50 |
| **immunonoglobulin heavy chain variable region partial** | gi\|393009287 | 17 | 8 | 6 | 2 | 7 | 7 |  | 3 |  |  |  |  |  |  |  | 2 |  |  |  |  |  |  |  |  |  |  |  |  |  | 52 |
|  | gi\|393009271 | 24 | 7 |  |  | 8 | 5 |  |  |  |  |  |  |  |  |  |  |  |  |  |  |  |  |  |  |  |  |  |  |  | 44 |
|  | gi\|393009293 | 16 | 7 |  |  |  | 5 |  | 3 |  |  |  |  |  |  |  |  |  |  |  |  |  |  |  |  |  |  |  |  |  | 31 |
| **immunoglobulin J chain** | gi\|697518601 | 16 | 2 | 1 |  | 2 | 5 |  | 1 |  |  |  |  |  |  |  |  |  |  |  |  |  |  |  |  |  |  |  |  |  | 27 |
| **immunoglobulin A heavy chain constant region transmemebrane form partial** | gi\|375162650 |  |  |  |  | 13 |  | 4 |  | 1 |  | 2 | 1 |  | 5 | 2 |  |  |  |  |  |  |  |  |  |  |  |  |  |  | 28 |
| **Immunoglobulin J chain partial** | gi\|678220694 | 16 | 2 | 1 |  | 2 | 5 |  | 1 |  |  |  |  |  |  |  | 1 |  |  |  |  |  |  |  |  |  |  |  |  |  | 28 |
| **immunonoglobulin heavy chain variable region partial** | gi\|393009285 | 15 |  |  |  | 5 |  |  | 3 |  |  |  |  |  |  |  |  |  |  |  |  |  |  |  |  |  |  |  |  |  | 23 |
| **immunonoglobulin heavy chain variable region partial** | gi\|393009295 | 15 |  |  |  | 5 |  |  | 3 |  |  |  |  |  |  |  |  |  |  |  |  |  |  |  |  |  |  |  |  |  | 23 |
| **immunonoglobulin heavy chain variable region partial** | gi\|393009281 | 16 |  |  |  |  |  |  | 3 |  |  |  |  |  |  |  | 2 |  |  |  |  |  |  |  |  |  |  |  |  |  | 21 |
| **immunoglobulin lambda variable region 12 partial** | gi\|375162678 | 9 |  | 2 |  |  | 5 |  | 2 |  |  |  |  |  |  |  |  |  |  |  |  |  |  |  |  |  |  |  |  |  | 18 |
| **ovostatin-like** | gi\|697505689 | 20 | 2 | 13 |  |  | 17 |  |  |  |  |  |  |  |  |  |  |  |  |  |  |  |  |  |  |  |  |  |  |  | 52 |
| **LOW QUALITY PROTEIN: hyaluronan and proteoglycan link protein 3 partial** | gi\|697502073 | 12 | 4 | 11 | 2 |  | 10 | 1 | 5 |  |  |  |  |  |  |  |  |  |  |  |  |  |  |  |  |  |  |  |  |  | 45 |
| **ovalbumin {ECO:0000303\|PubMed:21058653}** | gi\|697492053 | 21 |  | 17 |  |  | 3 |  |  |  |  |  |  |  |  |  |  |  |  |  |  |  |  |  |  |  |  |  |  |  | 41 |
| **serotriflin-like** | gi\|697488611 | 39 |  |  |  |  |  |  |  |  |  |  |  |  |  |  |  |  |  |  |  |  |  |  |  |  |  |  |  |  | 39 |
| **Neuroserpin** | gi\|678211082 | 9 | 5 | 5 |  | 3 | 4 | 0 | 3 |  |  |  |  |  |  |  |  |  |  |  |  |  |  |  |  |  |  |  |  |  | 29 |
| **neuroserpin** | gi\|697459011 | 9 | 5 | 5 |  | 3 | 4 | 0 | 3 |  |  |  |  |  |  |  |  |  |  |  |  |  |  |  |  |  |  |  |  |  | 29 |
| **prosaposin** | gi\|697432918 | 26 |  | 2 |  |  |  |  |  |  |  |  |  |  |  |  |  |  |  |  |  |  |  |  |  |  |  |  |  |  | 28 |
| **tetraspanin-1** | gi\|697481095 | 13 | 2 | 5 |  | 2 | 4 | 1 | 1 |  |  |  |  |  |  |  |  |  |  |  |  |  |  |  |  |  |  |  |  |  | 28 |
|  | gi\|697481098 | 13 | 2 | 5 |  | 2 | 4 | 1 | 1 |  |  |  |  |  |  |  |  |  |  |  |  |  |  |  |  |  |  |  |  |  | 28 |
|  | gi\|697481100 | 13 | 2 | 5 |  | 2 | 4 | 1 | 1 |  |  |  |  |  |  |  |  |  |  |  |  |  |  |  |  |  |  |  |  |  | 28 |
| **Secreted frizzled-related protein 3 partial** | gi\|678215055 | 12 | 3 | 3 | 3 |  | 1 | 2 | 3 |  |  |  |  |  |  |  |  |  |  |  |  |  |  |  |  |  |  |  |  |  | 27 |
|  | gi\|697487088 | 12 | 3 | 3 | 3 |  | 1 | 2 | 3 |  |  |  |  |  |  |  |  |  |  |  |  |  |  |  |  |  |  |  |  |  | 27 |
| **Cygnin** | gi\|678210025 | 22 |  |  |  | 2 |  |  |  |  |  |  |  |  |  |  | 1 |  |  |  |  |  |  |  |  |  |  |  |  |  | 25 |
|  | gi\|678210026 | 22 |  |  |  | 2 |  |  |  |  |  |  |  |  |  |  | 1 |  |  |  |  |  |  |  |  |  |  |  |  |  | 25 |
| **LOW QUALITY PROTEIN: versican core protein** | gi\|697493374 |  | 3 |  |  |  | 3 | 4 | 3 | 4 |  | 2 |  |  |  | 4 |  |  |  |  |  |  |  |  |  |  |  |  |  |  | 23 |
| **Signal peptide CUB and EGF-like domain-containing protein 1 partial** | gi\|678216365 | 22 |  |  |  |  |  |  |  |  |  |  |  |  |  |  |  |  |  |  |  |  |  |  |  |  |  |  |  |  | 22 |
| **extracellular serine/threonine protein kinase FAM20C** | gi\|697438505 | 15 | 1 | 2 |  |  | 4 |  |  |  |  |  |  |  |  |  |  |  |  |  |  |  |  |  |  |  |  |  |  |  | 22 |
| **N-acetylglucosamine-6-sulfatase partial** | gi\|678212640 | 15 |  | 2 |  |  | 4 |  |  |  |  |  |  |  |  |  |  |  |  |  |  |  |  |  |  |  |  |  |  |  | 21 |
|  | gi\|697474106 | 15 |  | 2 |  |  | 4 |  |  |  |  |  |  |  |  |  |  |  |  |  |  |  |  |  |  |  |  |  |  |  | 21 |
| **lactadherin partial** | gi\|697502076 | 10 | 4 | 2 |  |  | 3 |  |  | 2 |  |  |  |  |  |  |  |  |  |  |  |  |  |  |  |  |  |  |  |  | 21 |
| **Ovostatin partial** | gi\|678218405 |  | 2 |  |  |  | 17 |  |  |  |  |  |  |  |  |  |  |  |  |  |  |  |  |  |  |  |  |  |  |  | 19 |
| **Legumain partial** | gi\|678207887 | 15 |  | 3 |  |  |  |  |  |  |  |  |  |  |  |  |  |  |  |  |  |  |  |  |  |  |  |  |  |  | 18 |
|  | gi\|697437515 | 15 |  | 3 |  |  |  |  |  |  |  |  |  |  |  |  |  |  |  |  |  |  |  |  |  |  |  |  |  |  | 18 |
| **Nucleobindin-2** | gi\|678211376 | 18 |  |  |  |  |  |  |  |  |  |  |  |  |  |  |  |  |  |  |  |  |  |  |  |  |  |  |  |  | 18 |
|  | gi\|697463967 | 18 |  |  |  |  |  |  |  |  |  |  |  |  |  |  |  |  |  |  |  |  |  |  |  |  |  |  |  |  | 18 |
| **Interleukin-1 receptor type 1 partial** | gi\|678209628 | 13 | 3 | 2 |  |  |  |  |  |  |  |  |  |  |  |  |  |  |  |  |  |  |  |  |  |  |  |  |  |  | 18 |
|  | gi\|697443239 | 13 | 3 | 2 |  |  |  |  |  |  |  |  |  |  |  |  |  |  |  |  |  |  |  |  |  |  |  |  |  |  | 18 |
|  | gi\|697443241 | 13 | 3 | 2 |  |  |  |  |  |  |  |  |  |  |  |  |  |  |  |  |  |  |  |  |  |  |  |  |  |  | 18 |
|  | gi\|697443243 | 13 | 3 | 2 |  |  |  |  |  |  |  |  |  |  |  |  |  |  |  |  |  |  |  |  |  |  |  |  |  |  | 18 |
|  | gi\|697443247 | 13 | 3 | 2 |  |  |  |  |  |  |  |  |  |  |  |  |  |  |  |  |  |  |  |  |  |  |  |  |  |  | 18 |
|  | gi\|697443251 | 13 | 3 | 2 |  |  |  |  |  |  |  |  |  |  |  |  |  |  |  |  |  |  |  |  |  |  |  |  |  |  | 18 |
|  | gi\|697443253 | 13 | 3 | 2 |  |  |  |  |  |  |  |  |  |  |  |  |  |  |  |  |  |  |  |  |  |  |  |  |  |  | 18 |
| **ovoinhibitor-like** | gi\|697514085 | 18 |  |  |  |  |  |  |  |  |  |  |  |  |  |  |  |  |  |  |  |  |  |  |  |  |  |  |  |  | 18 |
| **immunoglobulin lambda variable region 3 partial** | gi\|375162660 | 11 |  | 2 |  | 4 |  |  |  |  |  |  |  |  |  |  |  |  |  |  |  |  |  |  |  |  |  |  |  |  | 17 |
| **immunonoglobulin heavy chain variable region partial** | gi\|393009273 |  |  |  |  | 7 |  | 1 | 3 |  |  | 3 |  |  |  |  | 2 |  |  |  |  |  |  |  |  |  |  |  |  |  | 16 |
| **immunoglobulin lambda variable region 6 partial** | gi\|375162666 | 8 |  |  |  | 3 | 3 |  |  |  |  |  |  |  |  |  |  |  |  |  |  |  |  |  |  |  |  |  |  |  | 14 |
| **immunoglobulin lambda variable region 7 partial** | gi\|375162668 | 9 |  | 2 |  |  |  |  | 2 |  |  |  |  |  |  |  |  |  |  |  |  |  |  |  |  |  |  |  |  |  | 13 |
| **immunonoglobulin heavy chain variable region partial** | gi\|393009291 | 13 |  |  |  |  |  |  |  |  |  |  |  |  |  |  |  |  |  |  |  |  |  |  |  |  |  |  |  |  | 13 |
| **LOW QUALITY PROTEIN: macrophage mannose receptor 1-like** | gi\|697479495 |  |  |  | 4 |  |  | 3 | 2 | 2 |  | 1 | 1 |  | 2 |  | 1 | 1 |  |  |  |  |  |  |  |  |  |  |  |  | 17 |
| **insulin-like growth factor-binding protein 7** | gi\|697508742 | 15 |  |  |  |  | 2 |  |  |  |  |  |  |  |  |  |  |  |  |  |  |  |  |  |  |  |  |  |  |  | 17 |
| **Ceruloplasmin partial** | gi\|678209318 | 10 |  | 3 |  |  | 1 |  |  |  |  |  |  |  |  |  |  |  |  |  |  |  |  |  |  |  |  |  |  |  | 14 |
| **ceruloplasmin isoform X1** | gi\|697441930 | 10 |  | 3 |  |  | 1 |  |  |  |  |  |  |  |  |  |  |  |  |  |  |  |  |  |  |  |  |  |  |  | 14 |
| **ceruloplasmin isoform X2** | gi\|697441933 | 10 |  | 3 |  |  | 1 |  |  |  |  |  |  |  |  |  |  |  |  |  |  |  |  |  |  |  |  |  |  |  | 14 |
| **uncharacterized protein LOC104141643 partial** | gi\|697472161 | 8 |  |  |  | 4 |  |  |  |  |  |  |  |  |  |  | 2 |  |  |  |  |  |  |  |  |  |  |  |  |  | 14 |
| **deleted in malignant brain tumors 1 protein-like** | gi\|697483399 | 10 |  | 2 |  |  | 2 |  |  |  |  |  |  |  |  |  |  |  |  |  |  |  |  |  |  |  |  |  |  |  | 14 |
| **beta-actin** | gi\|661912886 | 5 |  |  |  |  | 1 | 2 |  |  |  |  |  |  |  |  |  |  | 5 |  |  |  |  |  |  |  |  |  |  |  | 13 |
| **Actin cytoplasmic 1 partial** | gi\|678208200 | 5 |  |  |  |  | 1 | 2 |  |  |  |  |  |  |  |  |  |  | 5 |  |  |  |  |  |  |  |  |  |  |  | 13 |
| **Actin cytoplasmic type 5** | gi\|678218290 | 5 |  |  |  |  | 1 | 2 |  |  |  |  |  |  |  |  |  |  | 5 |  |  |  |  |  |  |  |  |  |  |  | 13 |
| **actin cytoplasmic 1** | gi\|697438274 | 5 |  |  |  |  | 1 | 2 |  |  |  |  |  |  |  |  |  |  | 5 |  |  |  |  |  |  |  |  |  |  |  | 13 |
| **Cadherin-2 partial** | gi\|678221852 | 11 |  | 2 |  |  |  |  |  |  |  |  |  |  |  |  |  |  |  |  |  |  |  |  |  |  |  |  |  |  | 13 |
| **cadherin-2 partial** | gi\|697524805 | 11 |  | 2 |  |  |  |  |  |  |  |  |  |  |  |  |  |  |  |  |  |  |  |  |  |  |  |  |  |  | 13 |
| **papilin isoform X7** | gi\|697470483 | 5 |  | 7 |  |  | 1 |  |  |  |  |  |  |  |  |  |  |  |  |  |  |  |  |  |  |  |  |  |  |  | 13 |
| **cystatin-like partial** | gi\|697477950 | 6 |  | 1 |  |  | 6 |  |  |  |  |  |  |  |  |  |  |  |  |  |  |  |  |  |  |  |  |  |  |  | 13 |
| **augurin** | gi\|697479952 | 2 | 1 | 1 | 2 | 3 | 1 |  | 1 |  |  |  |  |  | 1 |  | 1 |  |  |  |  |  |  |  |  |  |  |  |  |  | 13 |
| **actin cytoplasmic type 5** | gi\|697505172 | 5 |  |  |  |  | 1 | 2 |  |  |  |  |  |  |  |  |  |  | 5 |  |  |  |  |  |  |  |  |  |  |  | 13 |
| **immunoglobulin lambda variable region 11 partial** | gi\|375162676 |  | 5 | 2 |  |  | 5 |  |  |  |  |  |  |  |  |  |  |  |  |  |  |  |  |  |  |  |  |  |  |  | 12 |
| **Bactericidal permeability-increasing protein partial** | gi\|678205753 | 3 | 3 | 1 |  |  | 5 |  |  |  |  |  |  |  |  |  |  |  |  |  |  |  |  |  |  |  |  |  |  |  | 12 |
|  | gi\|697430981 | 3 | 3 | 1 |  |  | 5 |  |  |  |  |  |  |  |  |  |  |  |  |  |  |  |  |  |  |  |  |  |  |  | 12 |
| **Neural proliferation differentiation and control protein 1 partial** | gi\|678210857 | 8 | 2 |  |  |  |  | 1 |  |  |  |  | 1 |  |  |  |  |  |  |  |  |  |  |  |  |  |  |  |  |  | 12 |
|  | gi\|697455912 | 8 | 2 |  |  |  |  | 1 |  |  |  |  | 1 |  |  |  |  |  |  |  |  |  |  |  |  |  |  |  |  |  | 12 |
| **LOW QUALITY PROTEIN: sulfhydryl oxidase 1 partial** | gi\|697477064 | 12 |  |  |  |  |  |  |  |  |  |  |  |  |  |  |  |  |  |  |  |  |  |  |  |  |  |  |  |  | 12 |
| **Neuronal pentraxin-2 partial** | gi\|697438456 | 10 |  |  |  |  | 1 |  |  |  |  |  |  |  |  |  |  |  |  |  |  |  |  |  |  |  |  |  |  |  | 11 |
|  | gi\|678208251 | 10 |  |  |  |  |  |  |  |  |  |  |  |  |  |  |  |  |  |  |  |  |  |  |  |  |  |  |  |  | 10 |
| **Extracellular superoxide dismutase [Cu-Zn] partial** | gi\|678213415 | 10 |  |  |  |  |  |  |  |  |  |  |  |  |  |  |  |  |  |  |  |  |  |  |  |  |  |  |  |  | 10 |
| **agrin** | gi\|697473859 | 10 |  |  |  |  |  |  |  |  |  |  |  |  |  |  |  |  |  |  |  |  |  |  |  |  |  |  |  |  | 10 |
| **extracellular superoxide dismutase [Cu-Zn]** | gi\|697478397 | 10 |  |  |  |  |  |  |  |  |  |  |  |  |  |  |  |  |  |  |  |  |  |  |  |  |  |  |  |  | 10 |
|  | gi\|697478400 | 10 |  |  |  |  |  |  |  |  |  |  |  |  |  |  |  |  |  |  |  |  |  |  |  |  |  |  |  |  | 10 |
| **vascular non-inflammatory molecule 3-like partial** | gi\|697523171 | 9 |  |  |  |  |  |  |  |  |  |  |  |  | 1 |  |  |  |  |  |  |  |  |  |  |  |  |  |  |  | 10 |
| **78 kDa glucose-regulated protein partial** | gi\|678215743 | 9 |  |  |  |  |  |  |  |  |  |  |  |  |  |  |  |  |  |  |  |  |  |  |  |  |  |  |  |  | 9 |
|  | gi\|697490882 | 9 |  |  |  |  |  |  |  |  |  |  |  |  |  |  |  |  |  |  |  |  |  |  |  |  |  |  |  |  | 9 |
| **Actin aortic smooth muscle** | gi\|678215843 | 5 |  | 3 |  |  | 1 |  |  |  |  |  |  |  |  |  |  |  |  |  |  |  |  |  |  |  |  |  |  |  | 9 |
|  | gi\|697491469 | 5 |  | 3 |  |  | 1 |  |  |  |  |  |  |  |  |  |  |  |  |  |  |  |  |  |  |  |  |  |  |  | 9 |
|  | gi\|697491471 | 5 |  | 3 |  |  | 1 |  |  |  |  |  |  |  |  |  |  |  |  |  |  |  |  |  |  |  |  |  |  |  | 9 |
|  | gi\|697491473 | 5 |  | 3 |  |  | 1 |  |  |  |  |  |  |  |  |  |  |  |  |  |  |  |  |  |  |  |  |  |  |  | 9 |
| **Protein TENP partial** | gi\|678205749 | 4 |  |  |  |  |  |  | 3 | 1 |  |  |  |  |  |  |  |  |  |  |  |  |  |  |  |  |  |  |  |  | 8 |
| **Apolipoprotein A-I partial** | gi\|678211860 | 8 |  |  |  |  |  |  |  |  |  |  |  |  |  |  |  |  |  |  |  |  |  |  |  |  |  |  |  |  | 8 |
| **BPI fold-containing family B member 2** | gi\|697430969 | 4 |  |  |  |  |  |  | 3 | 1 |  |  |  |  |  |  |  |  |  |  |  |  |  |  |  |  |  |  |  |  | 8 |
| **papilin isoform X5** | gi\|697470474 |  |  | 7 |  |  | 1 |  |  |  |  |  |  |  |  |  |  |  |  |  |  |  |  |  |  |  |  |  |  |  | 8 |
| **papilin isoform X6** | gi\|697470478 |  |  | 7 |  |  | 1 |  |  |  |  |  |  |  |  |  |  |  |  |  |  |  |  |  |  |  |  |  |  |  | 8 |
| **papilin isoform X10** | gi\|697470492 |  |  | 7 |  |  | 1 |  |  |  |  |  |  |  |  |  |  |  |  |  |  |  |  |  |  |  |  |  |  |  | 8 |
| **ovostatin-like** | gi\|697505691 | 7 |  |  |  |  |  | 1 |  |  |  |  |  |  |  |  |  |  |  |  |  |  |  |  |  |  |  |  |  |  | 8 |
| **Follistatin-related protein 1 partial** | gi\|678207614 | 7 |  |  |  |  |  |  |  |  |  |  |  |  |  |  |  |  |  |  |  |  |  |  |  |  |  |  |  |  | 7 |
|  | gi\|697437240 | 7 |  |  |  |  |  |  |  |  |  |  |  |  |  |  |  |  |  |  |  |  |  |  |  |  |  |  |  |  | 7 |
| **Peptidyl-prolyl cis-trans isomerase B partial** | gi\|678210075 | 7 |  |  |  |  |  |  |  |  |  |  |  |  |  |  |  |  |  |  |  |  |  |  |  |  |  |  |  |  | 7 |
| **nephronectin** | gi\|697524624 | 7 |  |  |  |  |  |  |  |  |  |  |  |  |  |  |  |  |  |  |  |  |  |  |  |  |  |  |  |  | 7 |
| **Vitronectin partial** | gi\|678204965 | 6 |  |  |  |  |  |  |  |  |  |  |  |  |  |  |  |  |  |  |  |  |  |  |  |  |  |  |  |  | 6 |
| **Renin receptor partial** | gi\|678207298 | 6 |  |  |  |  |  |  |  |  |  |  |  |  |  |  |  |  |  |  |  |  |  |  |  |  |  |  |  |  | 6 |
| **EGF-containing fibulin-like extracellular matrix protein 1** | gi\|678211333 | 6 |  |  |  |  |  |  |  |  |  |  |  |  |  |  |  |  |  |  |  |  |  |  |  |  |  |  |  |  | 6 |
| **Draxin partial** | gi\|678212497 | 6 |  |  |  |  |  |  |  |  |  |  |  |  |  |  |  |  |  |  |  |  |  |  |  |  |  |  |  |  | 6 |
|  | gi\|697473255 | 6 |  |  |  |  |  |  |  |  |  |  |  |  |  |  |  |  |  |  |  |  |  |  |  |  |  |  |  |  | 6 |
|  | gi\|697473258 | 6 |  |  |  |  |  |  |  |  |  |  |  |  |  |  |  |  |  |  |  |  |  |  |  |  |  |  |  |  | 6 |
| **Receptor-type tyrosine-protein phosphatase F partial** | gi\|678217438 | 6 |  |  |  |  |  |  |  |  |  |  |  |  |  |  |  |  |  |  |  |  |  |  |  |  |  |  |  |  | 6 |
| **Actin alpha skeletal muscle B** | gi\|678221240 | 5 |  |  |  |  | 1 |  |  |  |  |  |  |  |  |  |  |  |  |  |  |  |  |  |  |  |  |  |  |  | 6 |
|  | gi\|697521731 | 5 |  |  |  |  | 1 |  |  |  |  |  |  |  |  |  |  |  |  |  |  |  |  |  |  |  |  |  |  |  | 6 |
| **EGF-containing fibulin-like extracellular matrix protein 1** | gi\|697462874 | 6 |  |  |  |  |  |  |  |  |  |  |  |  |  |  |  |  |  |  |  |  |  |  |  |  |  |  |  |  | 6 |
| **vitellogenin-2-like** | gi\|697478189 | 6 |  |  |  |  |  |  |  |  |  |  |  |  |  |  |  |  |  |  |  |  |  |  |  |  |  |  |  |  | 6 |
| **receptor-type tyrosine-protein phosphatase F** | gi\|697500216 | 6 |  |  |  |  |  |  |  |  |  |  |  |  |  |  |  |  |  |  |  |  |  |  |  |  |  |  |  |  | 6 |
| **protein FAM3C** | gi\|697518759 | 4 | 1 |  |  |  | 1 |  |  |  |  |  |  |  |  |  |  |  |  |  |  |  |  |  |  |  |  |  |  |  | 6 |
| **protein FAM3C** | gi\|697518761 | 4 | 1 |  |  |  | 1 |  |  |  |  |  |  |  |  |  |  |  |  |  |  |  |  |  |  |  |  |  |  |  | 6 |
| **uncharacterized protein LOC104152630** | gi\|697520723 | 4 | 1 | 1 |  |  |  |  |  |  |  |  |  |  |  |  |  |  |  |  |  |  |  |  |  |  |  |  |  |  | 6 |
| **antithrombin** | gi\|18140913 | 5 |  |  |  |  |  |  |  |  |  |  |  |  |  |  |  |  |  |  |  |  |  |  |  |  |  |  |  |  | 5 |
| **immunoglobulin Y heavy chain constant region secretory form partial** | gi\|375162652 | 5 |  |  |  |  |  |  |  |  |  |  |  |  |  |  |  |  |  |  |  |  |  |  |  |  |  |  |  |  | 5 |
| **immunoglobulin lambda variable region 5 partial** | gi\|375162664 | 5 |  |  |  |  |  |  |  |  |  |  |  |  |  |  |  |  |  |  |  |  |  |  |  |  |  |  |  |  | 5 |
| **Cadherin-1 partial** | gi\|678205435 | 5 |  |  |  |  |  |  |  |  |  |  |  |  |  |  |  |  |  |  |  |  |  |  |  |  |  |  |  |  | 5 |
| **Ovochymase-2 partial** | gi\|678206647 | 5 |  |  |  |  |  |  |  |  |  |  |  |  |  |  |  |  |  |  |  |  |  |  |  |  |  |  |  |  | 5 |
| **A disintegrin and metalloproteinase with thrombospondin motifs 5 partial** | gi\|678210638 | 5 |  |  |  |  |  |  |  |  |  |  |  |  |  |  |  |  |  |  |  |  |  |  |  |  |  |  |  |  | 5 |
| **Peptidyl-glycine alpha-amidating monooxygenase** | gi\|678211179 | 4 |  | 1 |  |  |  |  |  |  |  |  |  |  |  |  |  |  |  |  |  |  |  |  |  |  |  |  |  |  | 5 |
| **Alpha-enolase** | gi\|678212527 | 5 |  |  |  |  |  |  |  |  |  |  |  |  |  |  |  |  |  |  |  |  |  |  |  |  |  |  |  |  | 5 |
|  | gi\|697473395 | 5 |  |  |  |  |  |  |  |  |  |  |  |  |  |  |  |  |  |  |  |  |  |  |  |  |  |  |  |  | 5 |
| **Wnt inhibitory factor 1 partial** | gi\|678212637 | 2 |  | 1 |  |  | 2 |  |  |  |  |  |  |  |  |  |  |  |  |  |  |  |  |  |  |  |  |  |  |  | 5 |
| **Olfactomedin-4 partial** | gi\|678212966 | 5 |  |  |  |  |  |  |  |  |  |  |  |  |  |  |  |  |  |  |  |  |  |  |  |  |  |  |  |  | 5 |
| **Limbic system-associated membrane protein partial** | gi\|678214512 | 5 |  |  |  |  |  |  |  |  |  |  |  |  |  |  |  |  |  |  |  |  |  |  |  |  |  |  |  |  | 5 |
| **Vitelline membrane outer layer protein 1 partial** | gi\|678218938 |  |  |  |  |  |  |  |  |  |  |  |  | 5 |  |  |  |  |  |  |  |  |  |  |  |  |  |  |  |  | 5 |
| **Coagulation factor VIII partial** | gi\|678219487 | 5 |  |  |  |  |  |  |  |  |  |  |  |  |  |  |  |  |  |  |  |  |  |  |  |  |  |  |  |  | 5 |
| **LOW QUALITY PROTEIN: cadherin-1-like** | gi\|697429717 | 5 |  |  |  |  |  |  |  |  |  |  |  |  |  |  |  |  |  |  |  |  |  |  |  |  |  |  |  |  | 5 |
| **ovochymase-2** | gi\|697434076 | 5 |  |  |  |  |  |  |  |  |  |  |  |  |  |  |  |  |  |  |  |  |  |  |  |  |  |  |  |  | 5 |
| **peptidyl-prolyl cis-trans isomerase B** | gi\|697446146 | 5 |  |  |  |  |  |  |  |  |  |  |  |  |  |  |  |  |  |  |  |  |  |  |  |  |  |  |  |  | 5 |
| **A disintegrin and metalloproteinase with thrombospondin motifs 5 partial** | gi\|697451319 | 5 |  |  |  |  |  |  |  |  |  |  |  |  |  |  |  |  |  |  |  |  |  |  |  |  |  |  |  |  | 5 |
| **peptidyl-glycine alpha-amidating monooxygenase isoform X1** | gi\|697460341 | 4 |  | 1 |  |  |  |  |  |  |  |  |  |  |  |  |  |  |  |  |  |  |  |  |  |  |  |  |  |  | 5 |
| **peptidyl-glycine alpha-amidating monooxygenase isoform X1** | gi\|697460347 | 4 |  | 1 |  |  |  |  |  |  |  |  |  |  |  |  |  |  |  |  |  |  |  |  |  |  |  |  |  |  | 5 |
| **peptidyl-glycine alpha-amidating monooxygenase isoform X2** | gi\|697460351 | 4 |  | 1 |  |  |  |  |  |  |  |  |  |  |  |  |  |  |  |  |  |  |  |  |  |  |  |  |  |  | 5 |
| **peptidyl-glycine alpha-amidating monooxygenase isoform X3** | gi\|697460353 | 4 |  | 1 |  |  |  |  |  |  |  |  |  |  |  |  |  |  |  |  |  |  |  |  |  |  |  |  |  |  | 5 |
| **protein disulfide-isomerase** | gi\|697460798 | 5 |  |  |  |  |  |  |  |  |  |  |  |  |  |  |  |  |  |  |  |  |  |  |  |  |  |  |  |  | 5 |
| **papilin isoform X3** | gi\|697470458 | 5 |  |  |  |  |  |  |  |  |  |  |  |  |  |  |  |  |  |  |  |  |  |  |  |  |  |  |  |  | 5 |
| **antithrombin-III** | gi\|697476577 | 5 |  |  |  |  |  |  |  |  |  |  |  |  |  |  |  |  |  |  |  |  |  |  |  |  |  |  |  |  | 5 |
|  | gi\|697476580 | 5 |  |  |  |  |  |  |  |  |  |  |  |  |  |  |  |  |  |  |  |  |  |  |  |  |  |  |  |  | 5 |
|  | gi\|697476582 | 5 |  |  |  |  |  |  |  |  |  |  |  |  |  |  |  |  |  |  |  |  |  |  |  |  |  |  |  |  | 5 |
| **limbic system-associated membrane protein isoform X1** | gi\|697484005 | 5 |  |  |  |  |  |  |  |  |  |  |  |  |  |  |  |  |  |  |  |  |  |  |  |  |  |  |  |  | 5 |
| **limbic system-associated membrane protein isoform X2** | gi\|697484007 | 5 |  |  |  |  |  |  |  |  |  |  |  |  |  |  |  |  |  |  |  |  |  |  |  |  |  |  |  |  | 5 |
| **limbic system-associated membrane protein isoform X3** | gi\|697484009 | 5 |  |  |  |  |  |  |  |  |  |  |  |  |  |  |  |  |  |  |  |  |  |  |  |  |  |  |  |  | 5 |
| **alpha-fetoprotein-like isoform X1** | gi\|697508973 | 2 |  |  |  |  | 3 |  |  |  |  |  |  |  |  |  |  |  |  |  |  |  |  |  |  |  |  |  |  |  | 5 |
|  | gi\|697508975 | 2 |  |  |  |  | 3 |  |  |  |  |  |  |  |  |  |  |  |  |  |  |  |  |  |  |  |  |  |  |  | 5 |
|  | gi\|697508979 | 2 |  |  |  |  | 3 |  |  |  |  |  |  |  |  |  |  |  |  |  |  |  |  |  |  |  |  |  |  |  | 5 |
|  | gi\|697508981 | 2 |  |  |  |  | 3 |  |  |  |  |  |  |  |  |  |  |  |  |  |  |  |  |  |  |  |  |  |  |  | 5 |
| **alpha-fetoprotein-like isoform X2** | gi\|697508983 | 2 |  |  |  |  | 3 |  |  |  |  |  |  |  |  |  |  |  |  |  |  |  |  |  |  |  |  |  |  |  | 5 |
| **alpha-fetoprotein-like isoform X3** | gi\|697508985 | 2 |  |  |  |  | 3 |  |  |  |  |  |  |  |  |  |  |  |  |  |  |  |  |  |  |  |  |  |  |  | 5 |
| **alpha-fetoprotein-like isoform X4** | gi\|697508989 | 2 |  |  |  |  | 3 |  |  |  |  |  |  |  |  |  |  |  |  |  |  |  |  |  |  |  |  |  |  |  | 5 |
| **alpha-fetoprotein-like isoform X5** | gi\|697508991 | 2 |  |  |  |  | 3 |  |  |  |  |  |  |  |  |  |  |  |  |  |  |  |  |  |  |  |  |  |  |  | 5 |
| **coagulation factor VIII** | gi\|697512247 | 5 |  |  |  |  |  |  |  |  |  |  |  |  |  |  |  |  |  |  |  |  |  |  |  |  |  |  |  |  | 5 |
| **glutathione peroxidase 3** | gi\|697514149 | 5 |  |  |  |  |  |  |  |  |  |  |  |  |  |  |  |  |  |  |  |  |  |  |  |  |  |  |  |  | 5 |
| **prostatic acid phosphatase-like** | gi\|697520494 | 5 |  |  |  |  |  |  |  |  |  |  |  |  |  |  |  |  |  |  |  |  |  |  |  |  |  |  |  |  | 5 |
| **RecName: Full=Beta-microseminoprotein** | gi\|21263788 | 4 |  |  |  |  |  |  |  |  |  |  |  |  |  |  |  |  |  |  |  |  |  |  |  |  |  |  |  |  | 4 |
| **immunoglobulin lambda variable region 9 partial** | gi\|375162672 |  |  | 2 |  |  |  |  | 2 |  |  |  |  |  |  |  |  |  |  |  |  |  |  |  |  |  |  |  |  |  | 4 |
| **Dickkopf-related protein 3 partial** | gi\|678207213 | 4 |  |  |  |  |  |  |  |  |  |  |  |  |  |  |  |  |  |  |  |  |  |  |  |  |  |  |  |  | 4 |
| **Epithelial cell adhesion molecule partial** | gi\|678208264 | 4 |  |  |  |  |  |  |  |  |  |  |  |  |  |  |  |  |  |  |  |  |  |  |  |  |  |  |  |  | 4 |
| **Receptor-type tyrosine-protein phosphatase delta partial** | gi\|678208558 | 4 |  |  |  |  |  |  |  |  |  |  |  |  |  |  |  |  |  |  |  |  |  |  |  |  |  |  |  |  | 4 |
| **Neuronal pentraxin receptor partial** | gi\|678208737 | 4 |  |  |  |  |  |  |  |  |  |  |  |  |  |  |  |  |  |  |  |  |  |  |  |  |  |  |  |  | 4 |
| **hypothetical protein N308_07772** | gi\|678210268 | 4 |  |  |  |  |  |  |  |  |  |  |  |  |  |  |  |  |  |  |  |  |  |  |  |  |  |  |  |  | 4 |
| **Procollagen-lysine 2-oxoglutarate 5-dioxygenase 1 partial** | gi\|678212488 | 4 |  |  |  |  |  |  |  |  |  |  |  |  |  |  |  |  |  |  |  |  |  |  |  |  |  |  |  |  | 4 |
| **C1GALT1-specific chaperone 1 partial** | gi\|678212720 | 4 |  |  |  |  |  |  |  |  |  |  |  |  |  |  |  |  |  |  |  |  |  |  |  |  |  |  |  |  | 4 |
| **Ectonucleotide pyrophosphatase/phosphodiesterase family member 6 partial** | gi\|678215221 | 4 |  |  |  |  |  |  |  |  |  |  |  |  |  |  |  |  |  |  |  |  |  |  |  |  |  |  |  |  | 4 |
| **Beta-microseminoprotein partial** | gi\|678215867 | 4 |  |  |  |  |  |  |  |  |  |  |  |  |  |  |  |  |  |  |  |  |  |  |  |  |  |  |  |  | 4 |
| **Atrial natriuretic peptide-converting enzyme partial** | gi\|678217991 | 4 |  |  |  |  |  |  |  |  |  |  |  |  |  |  |  |  |  |  |  |  |  |  |  |  |  |  |  |  | 4 |
| **Glyceraldehyde-3-phosphate dehydrogenase partial** | gi\|678219539 | 4 |  |  |  |  |  |  |  |  |  |  |  |  |  |  |  |  |  |  |  |  |  |  |  |  |  |  |  |  | 4 |
| **Aminopeptidase N partial** | gi\|678220178 | 2 |  | 1 |  |  | 1 |  |  |  |  |  |  |  |  |  |  |  |  |  |  |  |  |  |  |  |  |  |  |  | 4 |
| **dickkopf-related protein 3** | gi\|697436187 | 4 |  |  |  |  |  |  |  |  |  |  |  |  |  |  |  |  |  |  |  |  |  |  |  |  |  |  |  |  | 4 |
| **epithelial cell adhesion molecule partial** | gi\|697438574 | 4 |  |  |  |  |  |  |  |  |  |  |  |  |  |  |  |  |  |  |  |  |  |  |  |  |  |  |  |  | 4 |
| **LOW QUALITY PROTEIN: receptor-type tyrosine-protein phosphatase delta-like** | gi\|697439379 | 4 |  |  |  |  |  |  |  |  |  |  |  |  |  |  |  |  |  |  |  |  |  |  |  |  |  |  |  |  | 4 |
| **neuronal pentraxin receptor-like partial** | gi\|697440259 | 4 |  |  |  |  |  |  |  |  |  |  |  |  |  |  |  |  |  |  |  |  |  |  |  |  |  |  |  |  | 4 |
| **GDNF family receptor alpha-1** | gi\|697459490 | 4 |  |  |  |  |  |  |  |  |  |  |  |  |  |  |  |  |  |  |  |  |  |  |  |  |  |  |  |  | 4 |
| **procollagen-lysine 2-oxoglutarate 5-dioxygenase 1** | gi\|697473817 | 4 |  |  |  |  |  |  |  |  |  |  |  |  |  |  |  |  |  |  |  |  |  |  |  |  |  |  |  |  | 4 |
| **C1GALT1-specific chaperone 1** | gi\|697474645 | 4 |  |  |  |  |  |  |  |  |  |  |  |  |  |  |  |  |  |  |  |  |  |  |  |  |  |  |  |  | 4 |
| **ectonucleotide pyrophosphatase/phosphodiesterase family member 6 isoform X1** | gi\|697487972 | 4 |  |  |  |  |  |  |  |  |  |  |  |  |  |  |  |  |  |  |  |  |  |  |  |  |  |  |  |  | 4 |
| **beta-microseminoprotein** | gi\|697491579 | 4 |  |  |  |  |  |  |  |  |  |  |  |  |  |  |  |  |  |  |  |  |  |  |  |  |  |  |  |  | 4 |
| **selenoprotein Pb-like** | gi\|697500265 | 4 |  |  |  |  |  |  |  |  |  |  |  |  |  |  |  |  |  |  |  |  |  |  |  |  |  |  |  |  | 4 |
| **atrial natriuretic peptide-converting enzyme-like** | gi\|697503279 | 4 |  |  |  |  |  |  |  |  |  |  |  |  |  |  |  |  |  |  |  |  |  |  |  |  |  |  |  |  | 4 |
| **proline-rich acidic protein 1** | gi\|697506889 | 4 |  |  |  |  |  |  |  |  |  |  |  |  |  |  |  |  |  |  |  |  |  |  |  |  |  |  |  |  | 4 |
| **glyceraldehyde-3-phosphate dehydrogenase** | gi\|697512486 | 4 |  |  |  |  |  |  |  |  |  |  |  |  |  |  |  |  |  |  |  |  |  |  |  |  |  |  |  |  | 4 |
| **aminopeptidase N** | gi\|697516043 | 2 |  | 1 |  |  | 1 |  |  |  |  |  |  |  |  |  |  |  |  |  |  |  |  |  |  |  |  |  |  |  | 4 |
| **fibulin-2 isoform X1** | gi\|697521054 | 4 |  |  |  |  |  |  |  |  |  |  |  |  |  |  |  |  |  |  |  |  |  |  |  |  |  |  |  |  | 4 |
| **fibulin-2 isoform X1** | gi\|697521058 | 4 |  |  |  |  |  |  |  |  |  |  |  |  |  |  |  |  |  |  |  |  |  |  |  |  |  |  |  |  | 4 |
| **fibulin-2 isoform X1** | gi\|697521061 | 4 |  |  |  |  |  |  |  |  |  |  |  |  |  |  |  |  |  |  |  |  |  |  |  |  |  |  |  |  | 4 |
| **fibulin-2 isoform X1** | gi\|697521064 | 4 |  |  |  |  |  |  |  |  |  |  |  |  |  |  |  |  |  |  |  |  |  |  |  |  |  |  |  |  | 4 |
| **fibulin-2 isoform X1** | gi\|697521066 | 4 |  |  |  |  |  |  |  |  |  |  |  |  |  |  |  |  |  |  |  |  |  |  |  |  |  |  |  |  | 4 |
| **fibulin-2 isoform X2** | gi\|697521068 | 4 |  |  |  |  |  |  |  |  |  |  |  |  |  |  |  |  |  |  |  |  |  |  |  |  |  |  |  |  | 4 |
| **immunoglobulin lambda variable region 8 partial** | gi\|375162670 | 3 |  |  |  |  |  |  |  |  |  |  |  |  |  |  |  |  |  |  |  |  |  |  |  |  |  |  |  |  | 3 |
| **Extracellular serine/threonine protein kinase FAM20C partial** | gi\|678208246 |  | 1 | 2 |  |  |  |  |  |  |  |  |  |  |  |  |  |  |  |  |  |  |  |  |  |  |  |  |  |  | 3 |
| **Gallinacin-12** | gi\|678210016 | 3 |  |  |  |  |  |  |  |  |  |  |  |  |  |  |  |  |  |  |  |  |  |  |  |  |  |  |  |  | 3 |
| **Ovocalyxin-32 partial** | gi\|678211070 | 3 |  |  |  |  |  |  |  |  |  |  |  |  |  |  |  |  |  |  |  |  |  |  |  |  |  |  |  |  | 3 |
| **C-type natriuretic peptide 1 partial** | gi\|678212492 | 3 |  |  |  |  |  |  |  |  |  |  |  |  |  |  |  |  |  |  |  |  |  |  |  |  |  |  |  |  | 3 |
| **C4b-binding protein alpha chain partial** | gi\|678212850 | 3 |  |  |  |  |  |  |  |  |  |  |  |  |  |  |  |  |  |  |  |  |  |  |  |  |  |  |  |  | 3 |
| **Cathepsin L1 partial** | gi\|678213372 | 3 |  |  |  |  |  |  |  |  |  |  |  |  |  |  |  |  |  |  |  |  |  |  |  |  |  |  |  |  | 3 |
| **Creatine kinase B-type** | gi\|678214649 | 3 |  |  |  |  |  |  |  |  |  |  |  |  |  |  |  |  |  |  |  |  |  |  |  |  |  |  |  |  | 3 |
| **Glia-derived nexin** | gi\|678214763 | 3 |  |  |  |  |  |  |  |  |  |  |  |  |  |  |  |  |  |  |  |  |  |  |  |  |  |  |  |  | 3 |
| **Beta-2-glycoprotein 1 partial** | gi\|678216478 | 3 |  |  |  |  |  |  |  |  |  |  |  |  |  |  |  |  |  |  |  |  |  |  |  |  |  |  |  |  | 3 |
| **Dystroglycan partial** | gi\|678217349 | 3 |  |  |  |  |  |  |  |  |  |  |  |  |  |  |  |  |  |  |  |  |  |  |  |  |  |  |  |  | 3 |
| **Heat shock cognate protein HSP 90-beta** | gi\|678220411 |  |  |  |  |  |  |  |  |  |  |  |  |  |  |  |  |  | 3 |  |  |  |  |  |  |  |  |  |  |  | 3 |
| **cadherin-like protein 26** | gi\|697431514 | 3 |  |  |  |  |  |  |  |  |  |  |  |  |  |  |  |  |  |  |  |  |  |  |  |  |  |  |  |  | 3 |
| **polyubiquitin-C** | gi\|697444235 |  |  | 1 |  |  |  |  |  |  |  |  |  |  |  |  |  |  |  |  |  |  |  |  | 1 |  |  | 1 |  |  | 3 |
| **ovocalyxin-32-like partial** | gi\|697459051 | 3 |  |  |  |  |  |  |  |  |  |  |  |  |  |  |  |  |  |  |  |  |  |  |  |  |  |  |  |  | 3 |
| **C-type natriuretic peptide** | gi\|697473234 | 3 |  |  |  |  |  |  |  |  |  |  |  |  |  |  |  |  |  |  |  |  |  |  |  |  |  |  |  |  | 3 |
| **signal peptide CUB and EGF-like domain-containing protein 3** | gi\|697474383 | 3 |  |  |  |  |  |  |  |  |  |  |  |  |  |  |  |  |  |  |  |  |  |  |  |  |  |  |  |  | 3 |
| **complement receptor type 1-like** | gi\|697475493 | 3 |  |  |  |  |  |  |  |  |  |  |  |  |  |  |  |  |  |  |  |  |  |  |  |  |  |  |  |  | 3 |
| **polyubiquitin-B** | gi\|697477409 |  |  | 1 |  |  |  |  |  |  |  |  |  |  |  |  |  |  |  |  |  |  |  |  | 1 |  |  | 1 |  |  | 3 |
| **cathepsin L1-like** | gi\|697478208 | 3 |  |  |  |  |  |  |  |  |  |  |  |  |  |  |  |  |  |  |  |  |  |  |  |  |  |  |  |  | 3 |
| **actin cytoplasmic 2-like** | gi\|697479256 |  |  |  |  |  | 1 | 2 |  |  |  |  |  |  |  |  |  |  |  |  |  |  |  |  |  |  |  |  |  |  | 3 |
| **creatine kinase B-type isoform X1** | gi\|697484858 | 3 |  |  |  |  |  |  |  |  |  |  |  |  |  |  |  |  |  |  |  |  |  |  |  |  |  |  |  |  | 3 |
| **creatine kinase B-type isoform X2** | gi\|697484863 | 3 |  |  |  |  |  |  |  |  |  |  |  |  |  |  |  |  |  |  |  |  |  |  |  |  |  |  |  |  | 3 |
| **glia-derived nexin isoform X1** | gi\|697485432 | 3 |  |  |  |  |  |  |  |  |  |  |  |  |  |  |  |  |  |  |  |  |  |  |  |  |  |  |  |  | 3 |
| **glia-derived nexin isoform X2** | gi\|697485434 | 3 |  |  |  |  |  |  |  |  |  |  |  |  |  |  |  |  |  |  |  |  |  |  |  |  |  |  |  |  | 3 |
| **delta and Notch-like epidermal growth factor-related receptor** | gi\|697485637 |  |  |  |  |  |  |  |  | 1 |  |  | 1 |  |  |  | 1 |  |  |  |  |  |  |  |  |  |  |  |  |  | 3 |
| **kininogen-1 isoform X1** | gi\|697489739 | 2 |  | 1 |  |  |  |  |  |  |  |  |  |  |  |  |  |  |  |  |  |  |  |  |  |  |  |  |  |  | 3 |
| **kininogen-1 isoform X2** | gi\|697489741 | 2 |  | 1 |  |  |  |  |  |  |  |  |  |  |  |  |  |  |  |  |  |  |  |  |  |  |  |  |  |  | 3 |
| **kininogen-1 isoform X3** | gi\|697489744 | 2 |  | 1 |  |  |  |  |  |  |  |  |  |  |  |  |  |  |  |  |  |  |  |  |  |  |  |  |  |  | 3 |
| **beta-2-glycoprotein 1** | gi\|697494710 | 3 |  |  |  |  |  |  |  |  |  |  |  |  |  |  |  |  |  |  |  |  |  |  |  |  |  |  |  |  | 3 |
| **dystroglycan** | gi\|697499556 | 3 |  |  |  |  |  |  |  |  |  |  |  |  |  |  |  |  |  |  |  |  |  |  |  |  |  |  |  |  | 3 |
| **protocadherin-16** | gi\|697504361 | 3 |  |  |  |  |  |  |  |  |  |  |  |  |  |  |  |  |  |  |  |  |  |  |  |  |  |  |  |  | 3 |
| **heat shock protein HSP 90-beta** | gi\|697516988 |  |  |  |  |  |  |  |  |  |  |  |  |  |  |  |  |  | 3 |  |  |  |  |  |  |  |  |  |  |  | 3 |
| **immunoglobulin lambda variable region 2 partial** | gi\|375162658 |  |  | 2 |  |  |  |  |  |  |  |  |  |  |  |  |  |  |  |  |  |  |  |  |  |  |  |  |  |  | 2 |
| **Gallinacin-10** | gi\|678204583 | 2 |  |  |  |  |  |  |  |  |  |  |  |  |  |  |  |  |  |  |  |  |  |  |  |  |  |  |  |  | 2 |
| **Ig heavy chain V-III region VH26 partial** | gi\|678204606 |  |  |  |  |  |  |  |  |  |  |  |  |  |  |  | 2 |  |  |  |  |  |  |  |  |  |  |  |  |  | 2 |
| **Elongation factor 1-alpha 2 partial** | gi\|678205919 |  |  |  |  |  |  |  |  |  |  |  |  |  |  |  |  |  | 2 |  |  |  |  |  |  |  |  |  |  |  | 2 |
| **Polypeptide N-acetylgalactosaminyltransferase 6 partial** | gi\|678207283 | 2 |  |  |  |  |  |  |  |  |  |  |  |  |  |  |  |  |  |  |  |  |  |  |  |  |  |  |  |  | 2 |
| **Fibroblast growth factor 20 partial** | gi\|678209280 | 2 |  |  |  |  |  |  |  |  |  |  |  |  |  |  |  |  |  |  |  |  |  |  |  |  |  |  |  |  | 2 |
| **Cathepsin B** | gi\|678210018 | 2 |  |  |  |  |  |  |  |  |  |  |  |  |  |  |  |  |  |  |  |  |  |  |  |  |  |  |  |  | 2 |
| **Palmitoyl-protein thioesterase 1 partial** | gi\|678210916 | 2 |  |  |  |  |  |  |  |  |  |  |  |  |  |  |  |  |  |  |  |  |  |  |  |  |  |  |  |  | 2 |
| **Epididymal secretory protein E1 partial** | gi\|678212255 | 2 |  |  |  |  |  |  |  |  |  |  |  |  |  |  |  |  |  |  |  |  |  |  |  |  |  |  |  |  | 2 |
| **Amyloid beta A4 protein partial** | gi\|678212312 | 2 |  |  |  |  |  |  |  |  |  |  |  |  |  |  |  |  |  |  |  |  |  |  |  |  |  |  |  |  | 2 |
| **45 kDa calcium-binding protein** | gi\|678212540 | 2 |  |  |  |  |  |  |  |  |  |  |  |  |  |  |  |  |  |  |  |  |  |  |  |  |  |  |  |  | 2 |
| **von Willebrand factor A domain-containing protein 1 partial** | gi\|678212554 | 2 |  |  |  |  |  |  |  |  |  |  |  |  |  |  |  |  |  |  |  |  |  |  |  |  |  |  |  |  | 2 |
| **Carboxypeptidase D partial** | gi\|678213233 | 2 |  |  |  |  |  |  |  |  |  |  |  |  |  |  |  |  |  |  |  |  |  |  |  |  |  |  |  |  | 2 |
| **Annexin A1 isoform p37** | gi\|678213269 | 2 |  |  |  |  |  |  |  |  |  |  |  |  |  |  |  |  |  |  |  |  |  |  |  |  |  |  |  |  | 2 |
| **Di-N-acetylchitobiase partial** | gi\|678213360 | 2 |  |  |  |  |  |  |  |  |  |  |  |  |  |  |  |  |  |  |  |  |  |  |  |  |  |  |  |  | 2 |
| **Out at first protein partial** | gi\|678213851 | 2 |  |  |  |  |  |  |  |  |  |  |  |  |  |  |  |  |  |  |  |  |  |  |  |  |  |  |  |  | 2 |
| **Calumenin partial** | gi\|678215446 | 2 |  |  |  |  |  |  |  |  |  |  |  |  |  |  |  |  |  |  |  |  |  |  |  |  |  |  |  |  | 2 |
| **Protein CREG1 partial** | gi\|678218361 |  | 2 |  |  |  |  |  |  |  |  |  |  |  |  |  |  |  |  |  |  |  |  |  |  |  |  |  |  |  | 2 |
| **Interleukin-13 receptor subunit alpha-2 partial** | gi\|678219470 | 2 |  |  |  |  |  |  |  |  |  |  |  |  |  |  |  |  |  |  |  |  |  |  |  |  |  |  |  |  | 2 |
| **Fibroblast growth factor 9** | gi\|678220326 | 2 |  |  |  |  |  |  |  |  |  |  |  |  |  |  |  |  |  |  |  |  |  |  |  |  |  |  |  |  | 2 |
| **Elongation factor 1-alpha 1** | gi\|678221662 |  |  |  |  |  |  |  |  |  |  |  |  |  |  |  |  |  | 2 |  |  |  |  |  |  |  |  |  |  |  | 2 |
| **LOW QUALITY PROTEIN: beta-1 4-galactosyltransferase 3** | gi\|697430234 | 2 |  |  |  |  |  |  |  |  |  |  |  |  |  |  |  |  |  |  |  |  |  |  |  |  |  |  |  |  | 2 |
| **LOW QUALITY PROTEIN: elongation factor 1-alpha 2** | gi\|697431398 |  |  |  |  |  |  |  |  |  |  |  |  |  |  |  |  |  | 2 |  |  |  |  |  |  |  |  |  |  |  | 2 |
| **LOW QUALITY PROTEIN: polypeptide N-acetylgalactosaminyltransferase 6** | gi\|697436346 | 2 |  |  |  |  |  |  |  |  |  |  |  |  |  |  |  |  |  |  |  |  |  |  |  |  |  |  |  |  | 2 |
| **lysosomal alpha-mannosidase** | gi\|697436929 | 2 |  |  |  |  |  |  |  |  |  |  |  |  |  |  |  |  |  |  |  |  |  |  |  |  |  |  |  |  | 2 |
| **insulin-like growth factor-binding protein 2** | gi\|697437317 | 2 |  |  |  |  |  |  |  |  |  |  |  |  |  |  |  |  |  |  |  |  |  |  |  |  |  |  |  |  | 2 |
| **insulin-like growth factor-binding protein 2** | gi\|697437320 | 2 |  |  |  |  |  |  |  |  |  |  |  |  |  |  |  |  |  |  |  |  |  |  |  |  |  |  |  |  | 2 |
| **fibroblast growth factor 20** | gi\|697441912 | 2 |  |  |  |  |  |  |  |  |  |  |  |  |  |  |  |  |  |  |  |  |  |  |  |  |  |  |  |  | 2 |
| **cathepsin B** | gi\|697445572 | 2 |  |  |  |  |  |  |  |  |  |  |  |  |  |  |  |  |  |  |  |  |  |  |  |  |  |  |  |  | 2 |
| **A disintegrin and metalloproteinase with thrombospondin motifs 1** | gi\|697451321 | 2 |  |  |  |  |  |  |  |  |  |  |  |  |  |  |  |  |  |  |  |  |  |  |  |  |  |  |  |  | 2 |
| **palmitoyl-protein thioesterase 1 partial** | gi\|697456492 | 2 |  |  |  |  |  |  |  |  |  |  |  |  |  |  |  |  |  |  |  |  |  |  |  |  |  |  |  |  | 2 |
| **alpha-N-acetylgalactosaminide alpha-2 6-sialyltransferase 2** | gi\|697458253 | 2 |  |  |  |  |  |  |  |  |  |  |  |  |  |  |  |  |  |  |  |  |  |  |  |  |  |  |  |  | 2 |
| **vitellogenin-1-like** | gi\|697471542 | 2 |  |  |  |  |  |  |  |  |  |  |  |  |  |  |  |  |  |  |  |  |  |  |  |  |  |  |  |  | 2 |
| **epididymal secretory protein E1** | gi\|697471949 | 2 |  |  |  |  |  |  |  |  |  |  |  |  |  |  |  |  |  |  |  |  |  |  |  |  |  |  |  |  | 2 |
| **amyloid beta A4 protein isoform X3** | gi\|697472043 | 2 |  |  |  |  |  |  |  |  |  |  |  |  |  |  |  |  |  |  |  |  |  |  |  |  |  |  |  |  | 2 |
| **amyloid beta A4 protein isoform X4** | gi\|697472046 | 2 |  |  |  |  |  |  |  |  |  |  |  |  |  |  |  |  |  |  |  |  |  |  |  |  |  |  |  |  | 2 |
| **45 kDa calcium-binding protein isoform X1** | gi\|697473446 | 2 |  |  |  |  |  |  |  |  |  |  |  |  |  |  |  |  |  |  |  |  |  |  |  |  |  |  |  |  | 2 |
| **45 kDa calcium-binding protein isoform X1** | gi\|697473448 | 2 |  |  |  |  |  |  |  |  |  |  |  |  |  |  |  |  |  |  |  |  |  |  |  |  |  |  |  |  | 2 |
| **45 kDa calcium-binding protein isoform X1** | gi\|697473451 | 2 |  |  |  |  |  |  |  |  |  |  |  |  |  |  |  |  |  |  |  |  |  |  |  |  |  |  |  |  | 2 |
| **45 kDa calcium-binding protein isoform X2** | gi\|697473455 | 2 |  |  |  |  |  |  |  |  |  |  |  |  |  |  |  |  |  |  |  |  |  |  |  |  |  |  |  |  | 2 |
| **45 kDa calcium-binding protein isoform X3** | gi\|697473458 | 2 |  |  |  |  |  |  |  |  |  |  |  |  |  |  |  |  |  |  |  |  |  |  |  |  |  |  |  |  | 2 |
| **von Willebrand factor A domain-containing protein 1** | gi\|697473541 | 2 |  |  |  |  |  |  |  |  |  |  |  |  |  |  |  |  |  |  |  |  |  |  |  |  |  |  |  |  | 2 |
| **wnt inhibitory factor 1** | gi\|697474093 | 2 |  |  |  |  |  |  |  |  |  |  |  |  |  |  |  |  |  |  |  |  |  |  |  |  |  |  |  |  | 2 |
| **carboxypeptidase D** | gi\|697477550 | 2 |  |  |  |  |  |  |  |  |  |  |  |  |  |  |  |  |  |  |  |  |  |  |  |  |  |  |  |  | 2 |
| **annexin A1 isoform p37** | gi\|697477686 | 2 |  |  |  |  |  |  |  |  |  |  |  |  |  |  |  |  |  |  |  |  |  |  |  |  |  |  |  |  | 2 |
| **di-N-acetylchitobiase** | gi\|697478194 | 2 |  |  |  |  |  |  |  |  |  |  |  |  |  |  |  |  |  |  |  |  |  |  |  |  |  |  |  |  | 2 |
| **out at first protein homolog** | gi\|697480583 | 2 |  |  |  |  |  |  |  |  |  |  |  |  |  |  |  |  |  |  |  |  |  |  |  |  |  |  |  |  | 2 |
| **out at first protein homolog** | gi\|697480585 | 2 |  |  |  |  |  |  |  |  |  |  |  |  |  |  |  |  |  |  |  |  |  |  |  |  |  |  |  |  | 2 |
| **calumenin isoform X1** | gi\|697489051 | 2 |  |  |  |  |  |  |  |  |  |  |  |  |  |  |  |  |  |  |  |  |  |  |  |  |  |  |  |  | 2 |
| **calumenin isoform X2** | gi\|697489053 | 2 |  |  |  |  |  |  |  |  |  |  |  |  |  |  |  |  |  |  |  |  |  |  |  |  |  |  |  |  | 2 |
| **alpha-2-HS-glycoprotein** | gi\|697489731 | 2 |  |  |  |  |  |  |  |  |  |  |  |  |  |  |  |  |  |  |  |  |  |  |  |  |  |  |  |  | 2 |
| **collagen alpha-1(VII) chain-like isoform X1** | gi\|697495610 | 2 |  |  |  |  |  |  |  |  |  |  |  |  |  |  |  |  |  |  |  |  |  |  |  |  |  |  |  |  | 2 |
| **collagen alpha-1(VII) chain-like isoform X1** | gi\|697495613 | 2 |  |  |  |  |  |  |  |  |  |  |  |  |  |  |  |  |  |  |  |  |  |  |  |  |  |  |  |  | 2 |
| **collagen alpha-1(VII) chain-like isoform X1** | gi\|697495615 | 2 |  |  |  |  |  |  |  |  |  |  |  |  |  |  |  |  |  |  |  |  |  |  |  |  |  |  |  |  | 2 |
| **collagen alpha-1(III) chain-like isoform X2** | gi\|697495617 | 2 |  |  |  |  |  |  |  |  |  |  |  |  |  |  |  |  |  |  |  |  |  |  |  |  |  |  |  |  | 2 |
| **basigin partial** | gi\|697498619 | 2 |  |  |  |  |  |  |  |  |  |  |  |  |  |  |  |  |  |  |  |  |  |  |  |  |  |  |  |  | 2 |
| **protein CREG1 partial** | gi\|697505407 |  | 2 |  |  |  |  |  |  |  |  |  |  |  |  |  |  |  |  |  |  |  |  |  |  |  |  |  |  |  | 2 |
| **LOW QUALITY PROTEIN: interleukin-13 receptor subunit alpha-2** | gi\|697512056 | 2 |  |  |  |  |  |  |  |  |  |  |  |  |  |  |  |  |  |  |  |  |  |  |  |  |  |  |  |  | 2 |
| **fibroblast growth factor 9** | gi\|697516611 | 2 |  |  |  |  |  |  |  |  |  |  |  |  |  |  |  |  |  |  |  |  |  |  |  |  |  |  |  |  | 2 |
| **melanotransferrin** | gi\|697523348 |  |  |  |  |  |  |  |  |  |  | 2 |  |  |  |  |  |  |  |  |  |  |  |  |  |  |  |  |  |  | 2 |
| **elongation factor 1-alpha 1** | gi\|697523715 |  |  |  |  |  |  |  |  |  |  |  |  |  |  |  |  |  | 2 |  |  |  |  |  |  |  |  |  |  |  | 2 |

| **Sequence Coverage (%)** | |
| --- | --- |
| 1 to 20 |  |
| 21 to 40 |  |
| 31 to 60 |  |
| 61 to 80 |  |
| 81 to 100 |  |

**Key: Numbers in cells are number of identified CID-induced product ion spectra; cell colour reflects sequence coverage.**
