# Supplementary material for: Protein sequences bound to mineral surfaces persist into deep time
Source: eLife. 2016 Sep 27;5:e17092. doi: 10.7554/eLife.17092 (PMC5039028; doi:10.7554/eLife.17092)
Supplement: Supplementary file 2. — Raw spectra (manually annotated on the basis of PEAKS assignments) of all the identified sequences identified in panel 1. 2–9: Copenhagen dataset; 10–26: York/Oxford dataset. DOI: http://dx.doi.org/10.7554/eLife.17092.021 [file elife-17092-supp2.pdf]

1

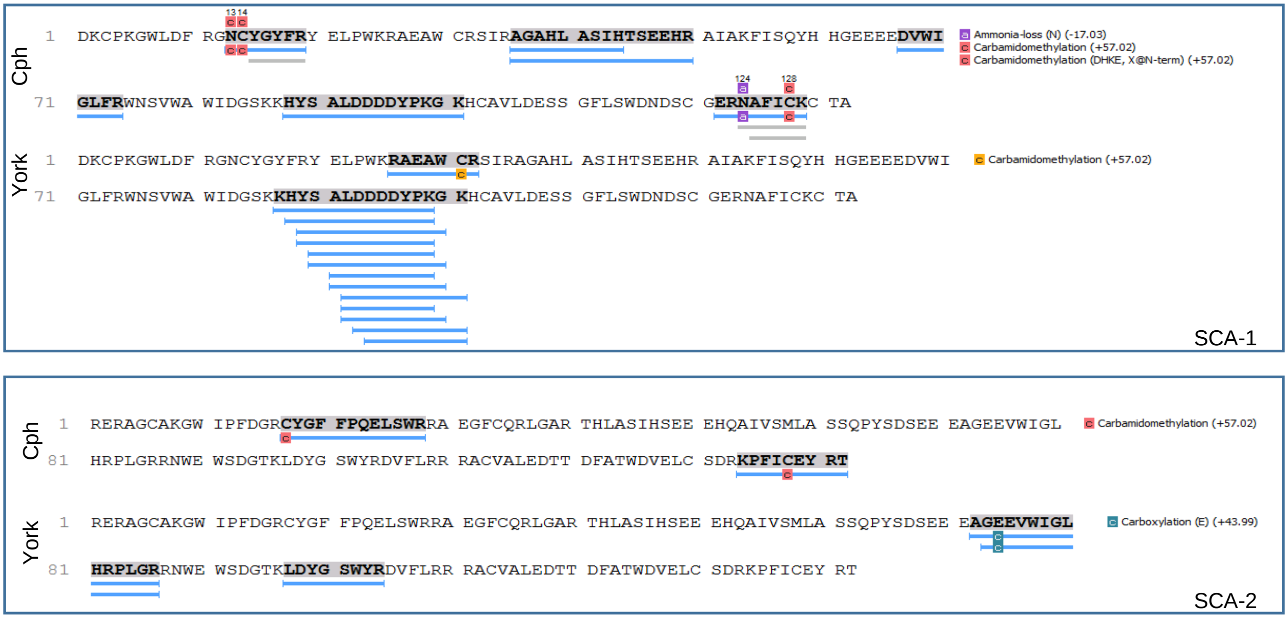
Cph
1 DKCPKGWLDF RGNCYGYFRY ELPWKRAEAW CRSIRAGAHL ASIHTSEEHR AIAKFISQYH HGEEEEDVWI
71 GLFRWNSVWA WIDGSKKHYS ALDDDDYPKG KHCAVLDESS GFLSWDNDSC GERNAFICKC TA
Ammonia-loss (N) (-17.03)
Carbamidomethylation (+57.02)
Carbamidomethylation (DHKE, X@N-term) (+57.02)
York
1 DKCPKGWLDF RGNCYGYFRY ELPWKRAEAW CRSIRAGAHL ASIHTSEEHR AIAKFISQYH HGEEEEDVWI
71 GLFRWNSVWA WIDGSKKHYS ALDDDDYPKG KHCAVLDESS GFLSWDNDSC GERNAFICKC TA
Carbamidomethylation (+57.02)
SCA-1
Cph
1 RERAGCAKGW IPFDGRCYGF FPQELSWRRA EGFCQRLGAR THLASIHSEE EHQAIVSMLA SSQPYSDSEE EAGEEVWIGL
81 HRPLGRRNWE WSDGTKLDYG SWYRDVFLRR RACVALEDTT DFATWDVELC SDRKPFICEY RT
Carbamidomethylation (+57.02)
York
1 RERAGCAKGW IPFDGRCYGF FPQELSWRRA EGFCQRLGAR THLASIHSEE EHQAIVSMLA SSQPYSDSEE EAGEEVWIGL
81 HRPLGRRNWE WSDGTKLDYG SWYRDVFLRR RACVALEDTT DFATWDVELC SDRKPFICEY RT
Carboxylation (E) (+43.99)
SCA-2

2

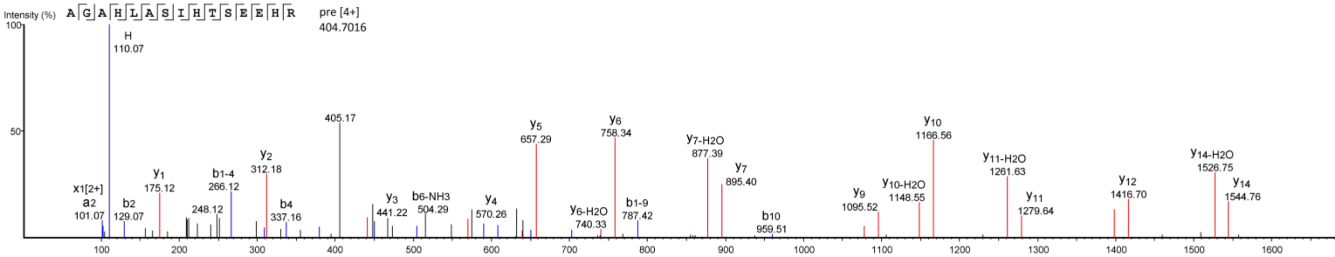
Intensity (%)
A G A H L A S I H T S E E H R
pre [4+]
404.7016
m/z

3

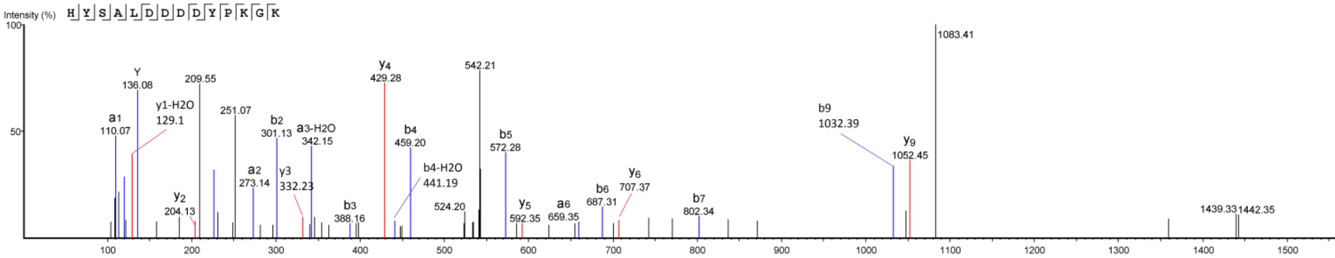
Intensity (%)
H Y S A L D D D D Y P K G K
m/z

4

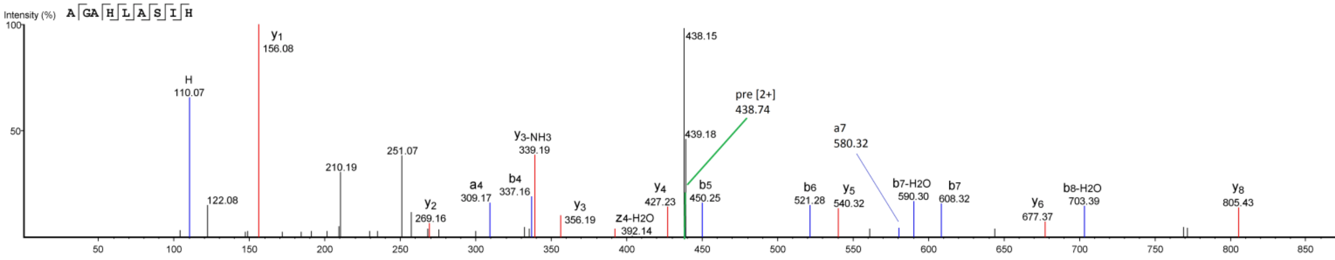
Intensity (%)
A GA H L A S I H
pre [2+]
438.74
m/z

5

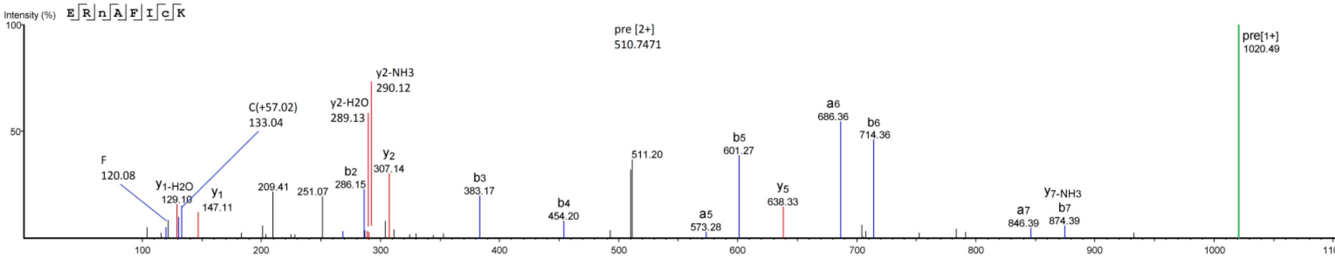
Intensity (%)
E R n A F I c K
pre [2+]
510.7471
pre[1+]
1020.49
m/z

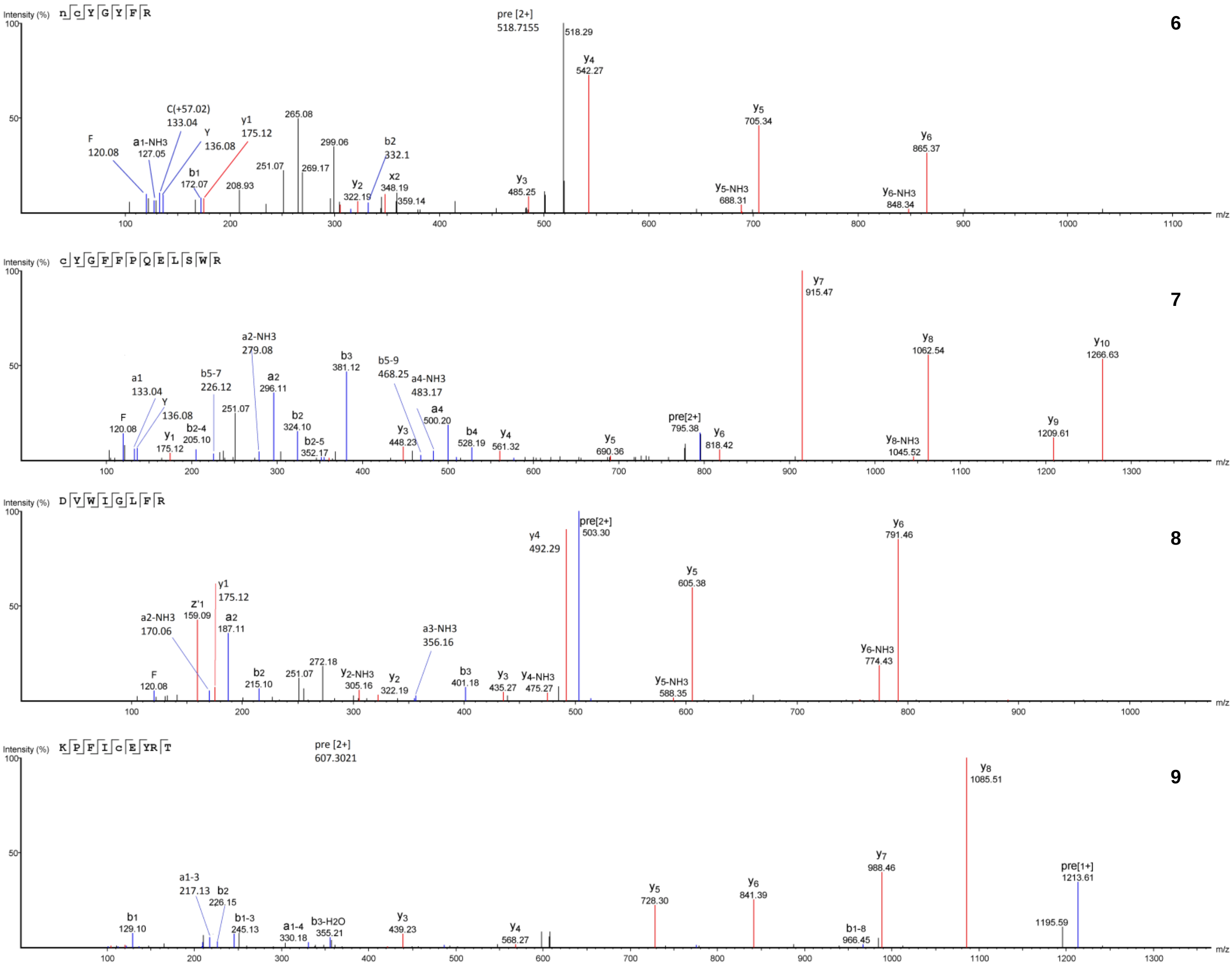

2-9: Product ion spectra (raw spectra manually annotated on the basis of PEAKS assignments) of all the Copenhagen identified sequences (see panel 1).

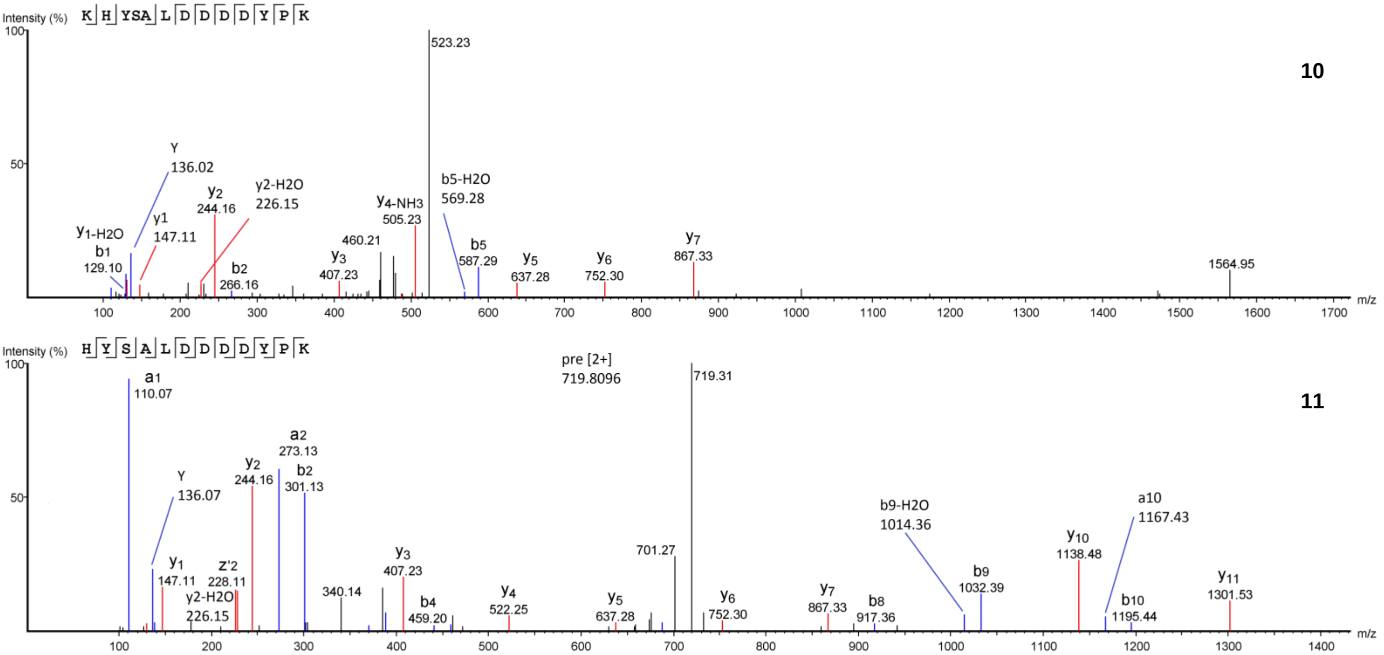

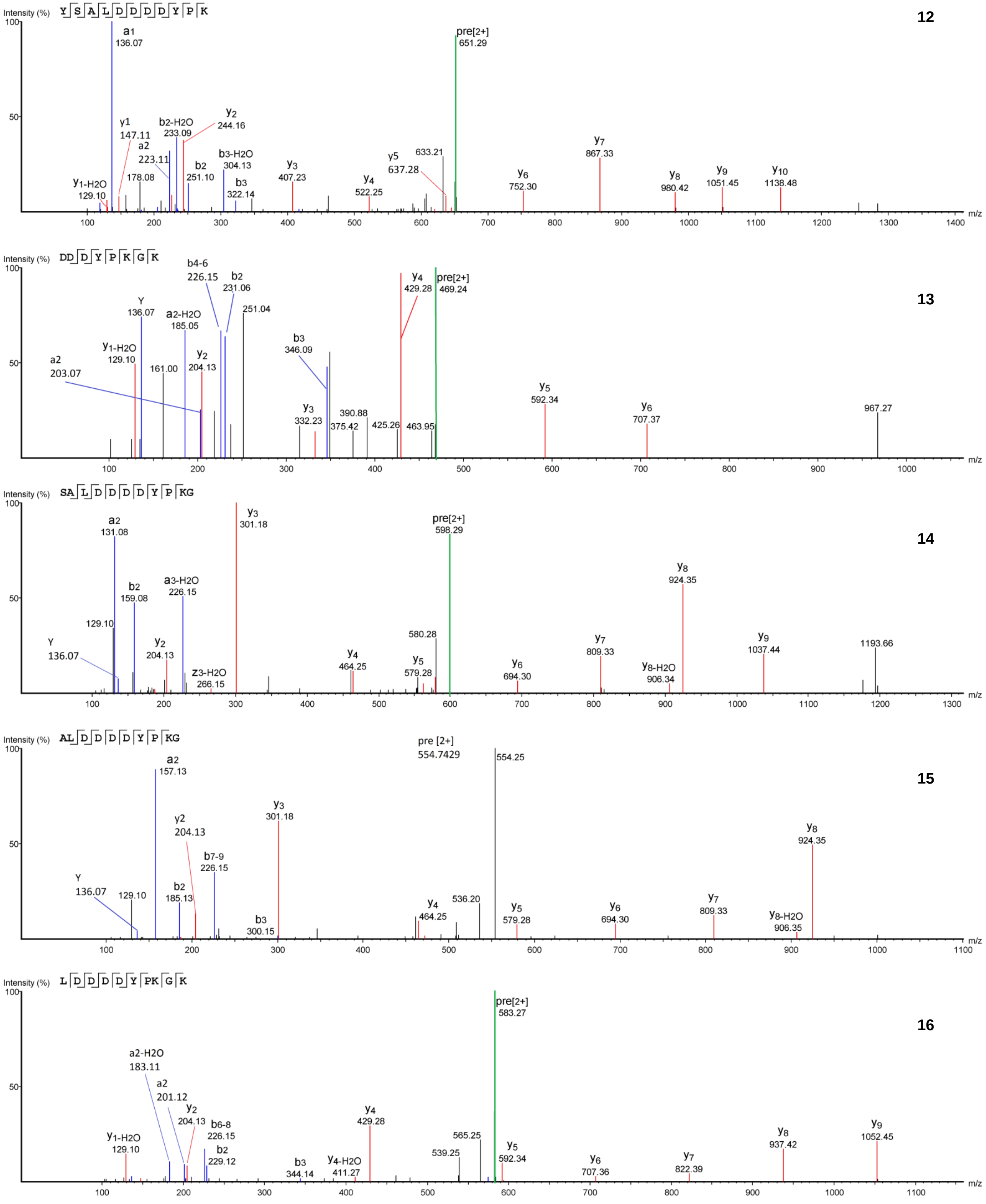

Intensity (%)
Y S A L D D D D Y P K
12
a1 136.07
pre[2+] 651.29
y1 147.11
b2-H2O 233.09
y2 244.16
a2 223.11
178.08
y1-H2O 129.10
b2 251.10
b3-H2O 304.13
b3 322.14
y3 407.23
y4 522.25
y5 637.28
633.21
y6 752.30
y7 867.33
y8 980.42
y9 1051.45
y10 1138.48
m/z
Intensity (%)
DD D Y P K G K
13
b4-6 226.15
b2 231.06
y4 429.28
pre[2+] 469.24
Y 136.07
a2-H2O 185.05
251.04
y1-H2O 129.10
a2 203.07
161.00
y2 204.13
b3 346.09
y3 332.23
390.88
375.42
425.26
463.95
y5 592.34
y6 707.37
967.27
m/z
Intensity (%)
SA L D D D D Y P KG
14
a2 131.08
y3 301.18
pre[2+] 598.29
b2 159.08
a3-H2O 226.15
129.10
Y 136.07
y2 204.13
z3-H2O 266.15
y4 464.25
580.28
y5 579.28
y6 694.30
y7 809.33
y8 924.35
y8-H2O 906.34
y9 1037.44
1193.66
m/z
Intensity (%)
AL D D D D Y P KG
15
pre [2+] 554.7429
554.25
a2 157.13
y3 301.18
y2 204.13
b7-9 226.15
Y 136.07
129.10
b2 185.13
b3 300.15
y4 464.25
536.20
y5 579.28
y6 694.30
y7 809.33
y8-H2O 906.35
y8 924.35
m/z
Intensity (%)
L D D D D Y PK G K
16
pre[2+] 583.27
a2-H2O 183.11
a2 201.12
y2 204.13
b6-8 226.15
b2 229.12
y1-H2O 129.10
y4 429.28
b3 344.14
y4-H2O 411.27
565.25
539.25
y5 592.34
y6 707.36
y7 822.39
y8 937.42
y9 1052.45
m/z

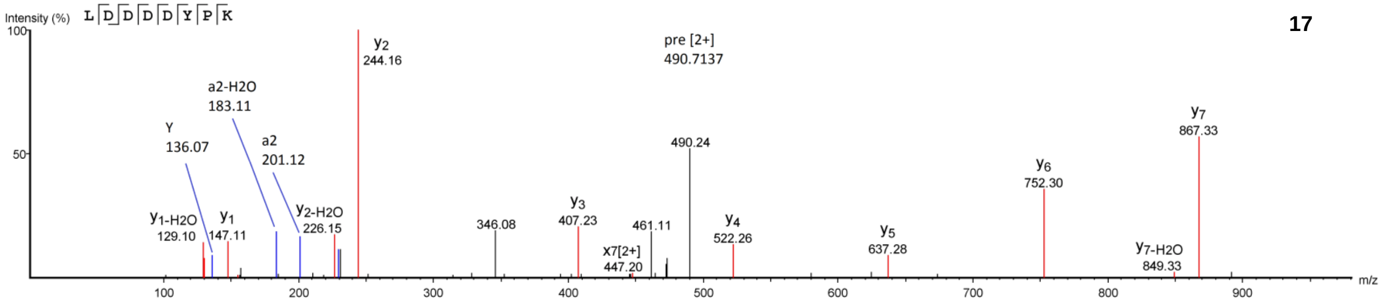
Intensity (%)
L D D D D Y P K
17
100
50
y2 244.16
pre [2+] 490.7137
a2-H2O 183.11
Y 136.07
a2 201.12
490.24
y7 867.33
y6 752.30
y1-H2O 129.10
y1 147.11
y2-H2O 226.15
346.08
y3 407.23
461.11
x7[2+] 447.20
y4 522.26
y5 637.28
y7-H2O 849.33
100 200 300 400 500 600 700 800 900
m/z

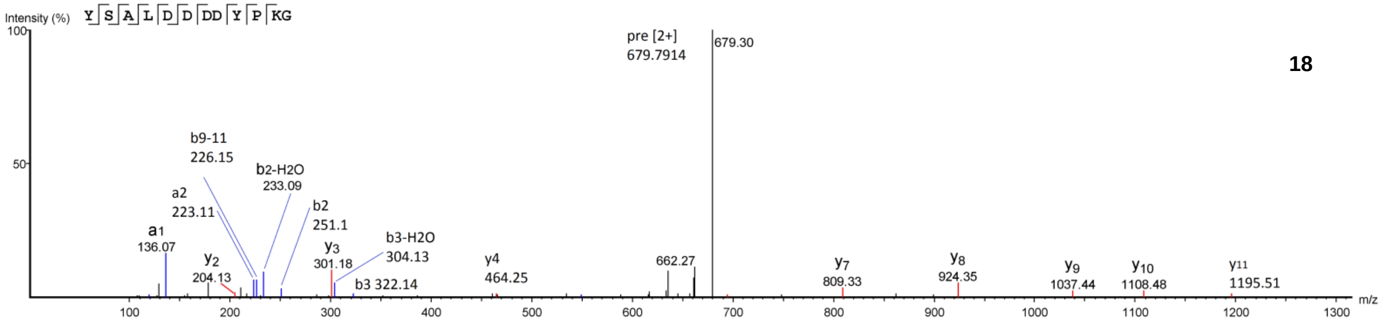
Intensity (%)
Y S A L D D DD Y P KG
18
100
50
pre [2+] 679.7914
679.30
b9-11 226.15
b2-H2O 233.09
a2 223.11
b2 251.1
a1 136.07
y3 301.18
b3-H2O 304.13
y2 204.13
b3 322.14
y4 464.25
662.27
y7 809.33
y8 924.35
y9 1037.44
y10 1108.48
y11 1195.51
100 200 300 400 500 600 700 800 900 1000 1100 1200 1300
m/z

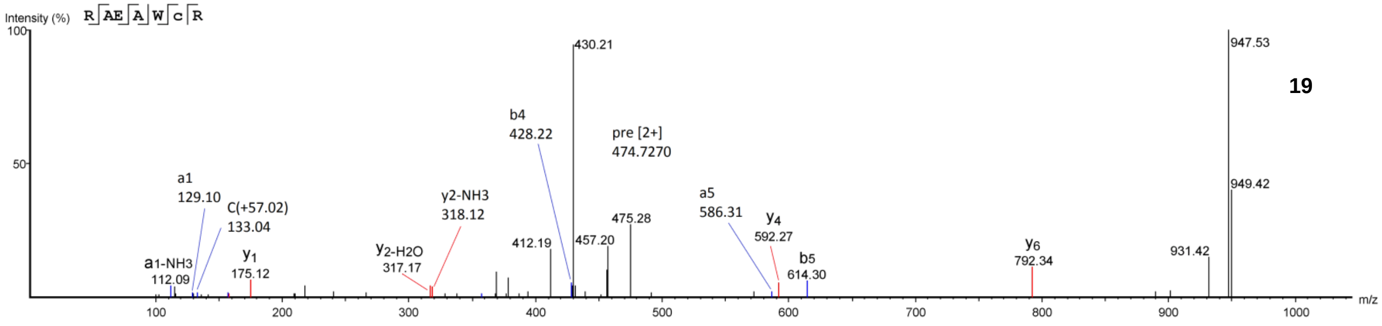
Intensity (%)
R AE A W c R
19
100
50
430.21
947.53
b4 428.22
pre [2+] 474.7270
949.42
a1 129.10
C(+57.02) 133.04
y2-NH3 318.12
475.28
a5 586.31
y4 592.27
a1-NH3 112.09
y1 175.12
y2-H2O 317.17
412.19
457.20
b5 614.30
y6 792.34
931.42
100 200 300 400 500 600 700 800 900 1000
m/z

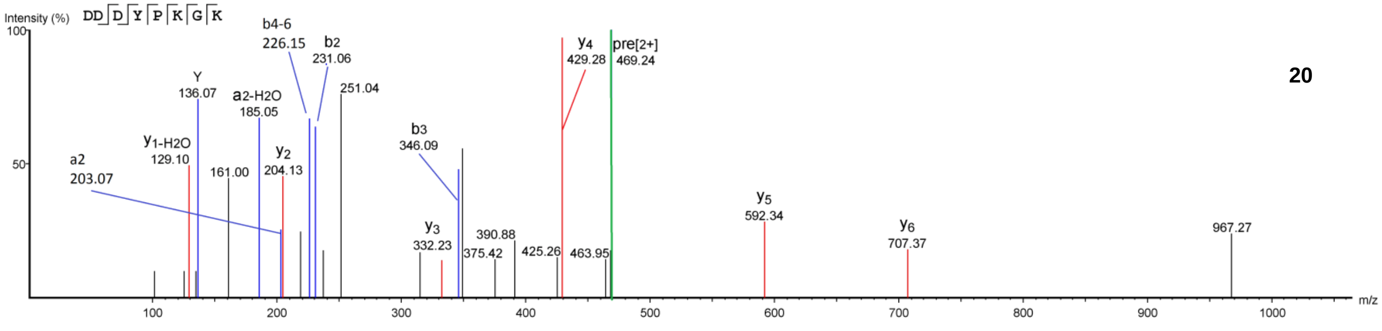
Intensity (%)
DD D Y P K G K
20
100
50
b4-6 226.15
b2 231.06
y4 429.28
pre[2+] 469.24
Y 136.07
a2-H2O 185.05
251.04
b3 346.09
y1-H2O 129.10
161.00
y2 204.13
a2 203.07
y5 592.34
y6 707.37
967.27
y3 332.23
390.88
375.42
425.26
463.95
100 200 300 400 500 600 700 800 900 1000
m/z

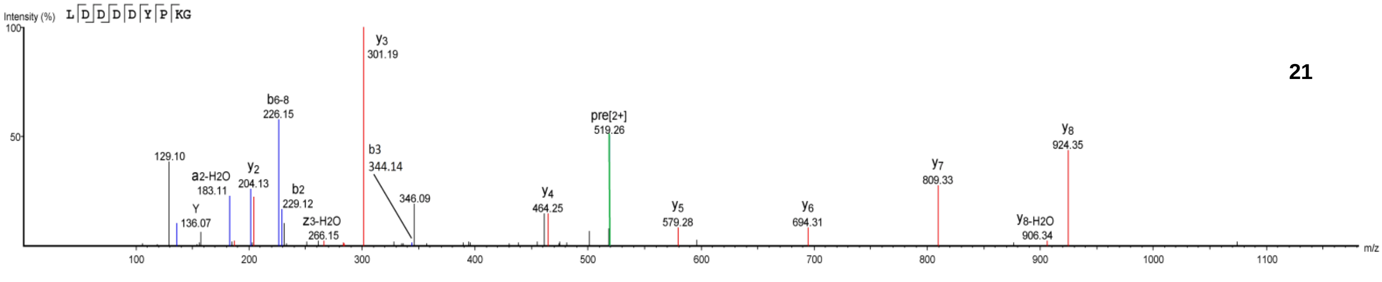
Intensity (%)
L D D D D Y P KG
21
100
50
y3 301.19
b6-8 226.15
pre[2+] 519.26
y8 924.35
129.10
b3 344.14
y7 809.33
a2-H2O 183.11
y2 204.13
b2 229.12
346.09
y4 464.25
Y 136.07
z3-H2O 266.15
y5 579.28
y6 694.31
y8-H2O 906.34
100 200 300 400 500 600 700 800 900 1000 1100
m/z

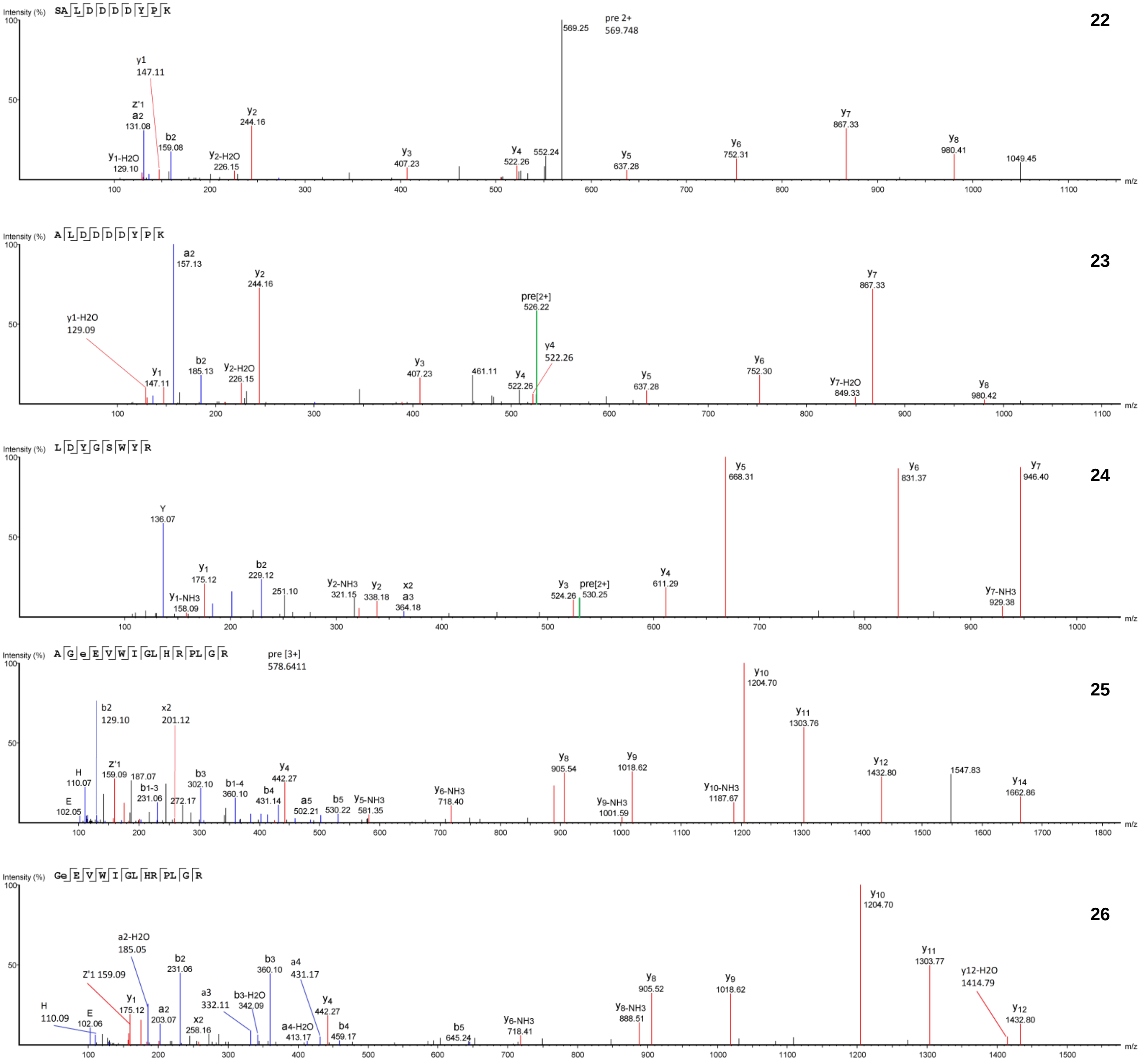

10-26: Product ion spectra (raw spectra manually annotated on the basis of PEAKS assignments) of all the York/Oxford identified sequences (see panel 1).
