# Supplementary material for: Protein sequences bound to mineral surfaces persist into deep time
Source: eLife. 2016 Sep 27;5:e17092. doi: 10.7554/eLife.17092 (PMC5039028; doi:10.7554/eLife.17092)
Supplement: Supplementary file 3. — Modifications detected in SCA-1 and SCA-2 sequences in all OES samples analysed. DOI: http://dx.doi.org/10.7554/eLife.17092.022 [file elife-17092-supp3.pdf]

1-17: Frequency of common modifications detected in each position of the sequence of SCA-1 in all bleached OES samples.

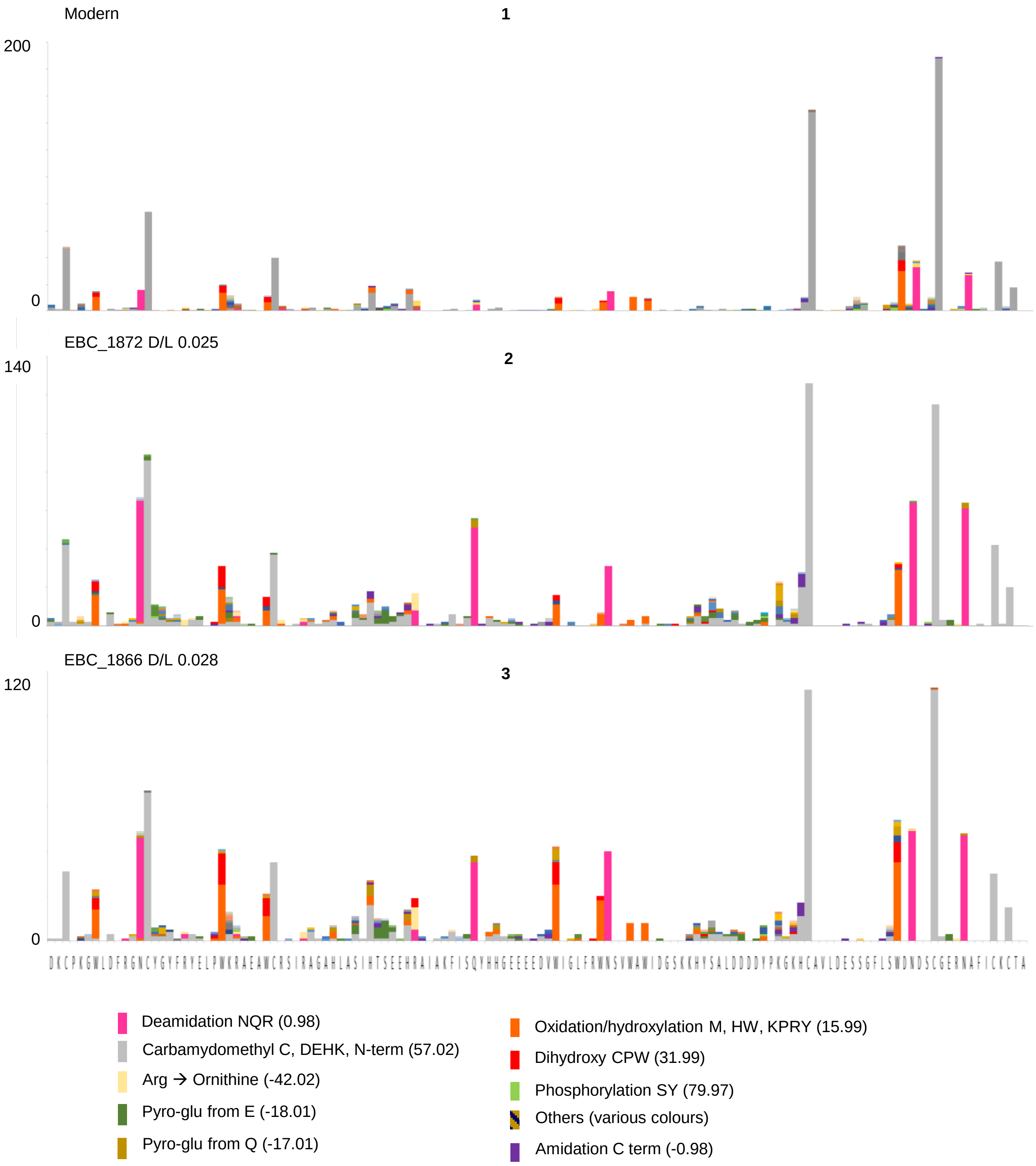

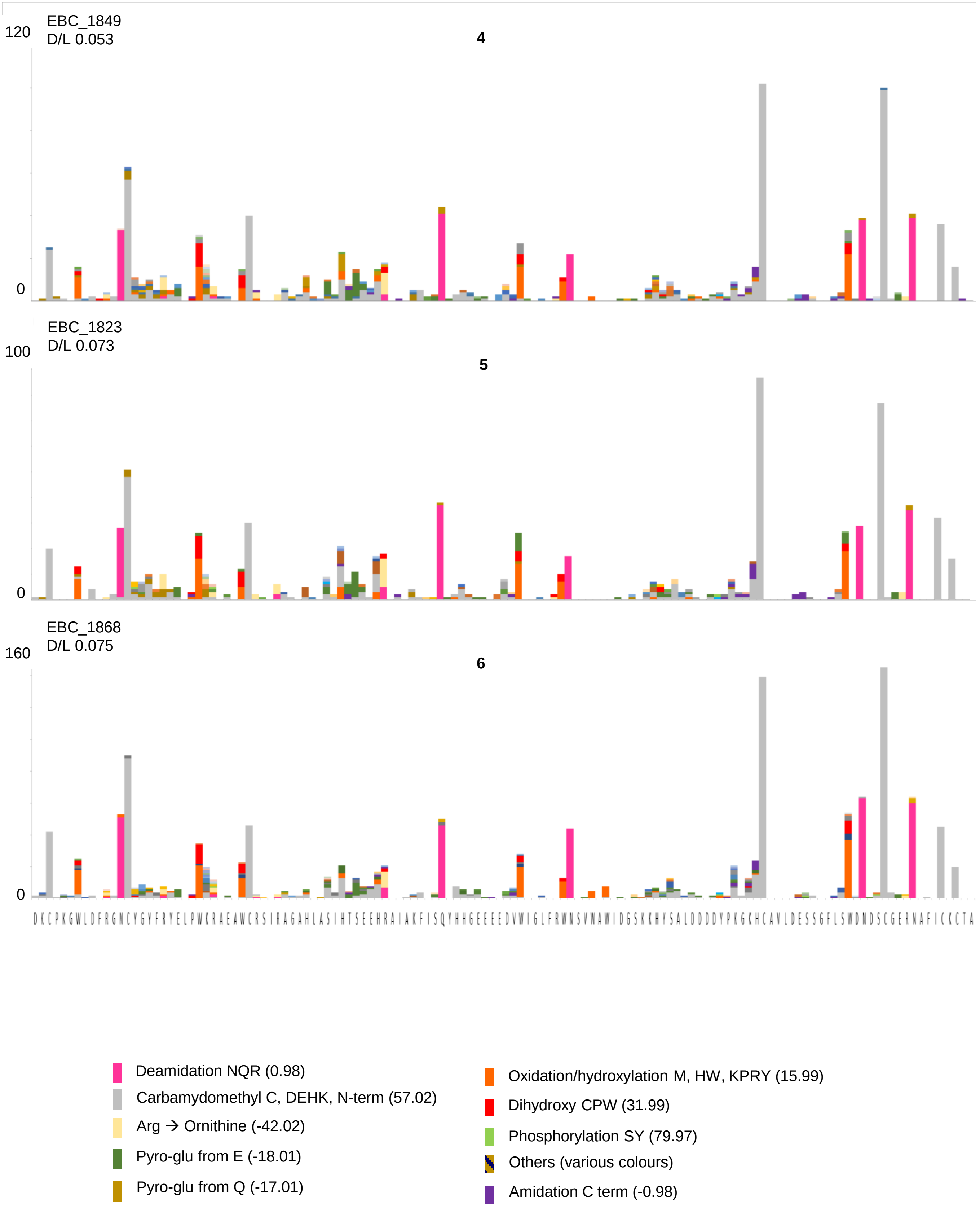

EBC_1849
D/L 0.053
120
0
4
EBC_1823
D/L 0.073
100
0
5
EBC_1868
D/L 0.075
160
0
6
Deamidation NQR (0.98)
Carbamydomethyl C, DEHK, N-term (57.02)
Arg → Ornithine (-42.02)
Pyro-glu from E (-18.01)
Pyro-glu from Q (-17.01)
Oxidation/hydroxylation M, HW, KPRY (15.99)
Dihydroxy CPW (31.99)
Phosphorylation SY (79.97)
Others (various colours)
Amidation C term (-0.98)

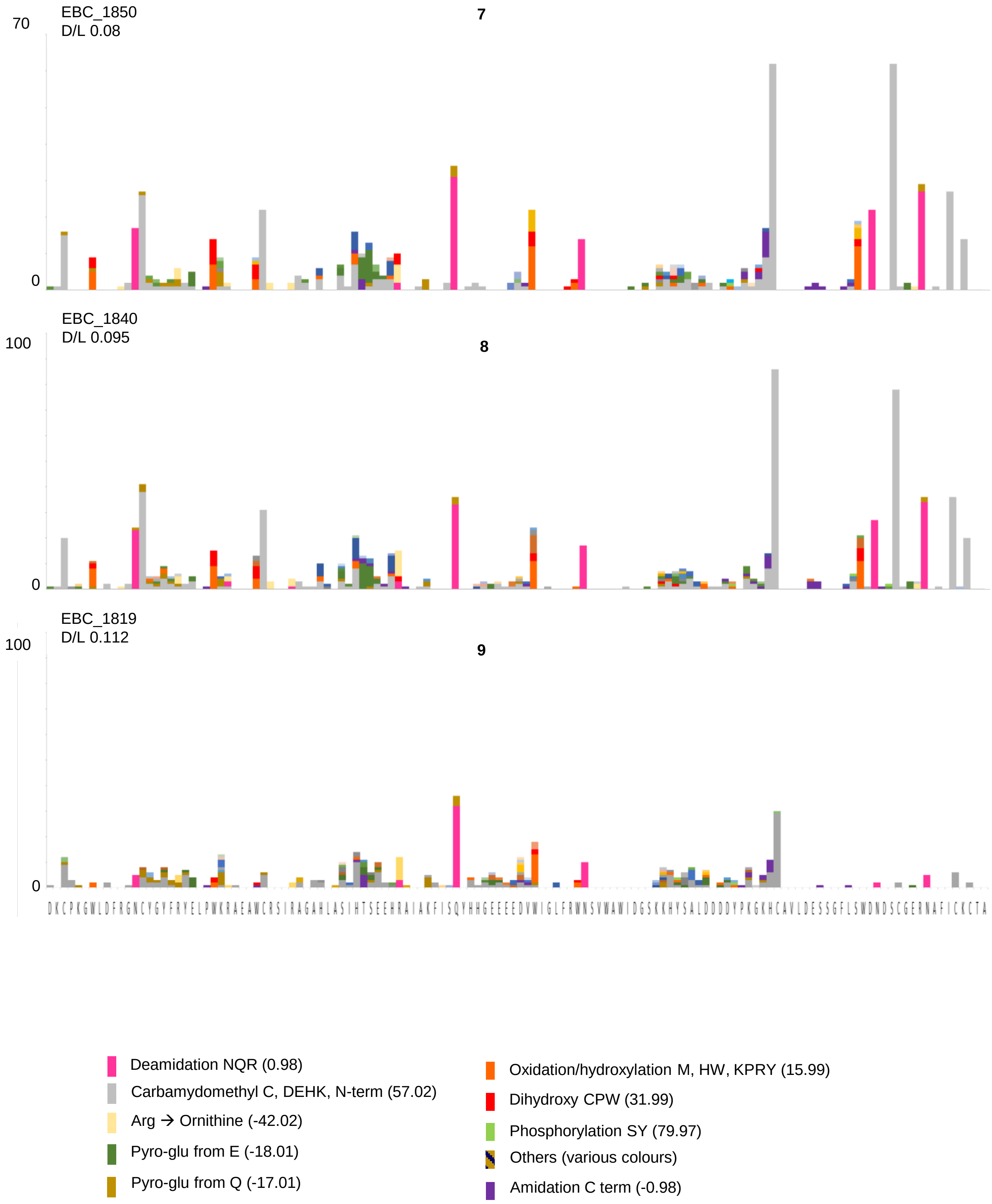
EBC_1850
D/L 0.08
7
70
0
EBC_1840
D/L 0.095
100
8
0
EBC_1819
D/L 0.112
100
9
0
Deamidation NQR (0.98)
Carbamydomethyl C, DEHK, N-term (57.02)
Arg → Ornithine (-42.02)
Pyro-glu from E (-18.01)
Pyro-glu from Q (-17.01)
Oxidation/hydroxylation M, HW, KPRY (15.99)
Dihydroxy CPW (31.99)
Phosphorylation SY (79.97)
Others (various colours)
Amidation C term (-0.98)

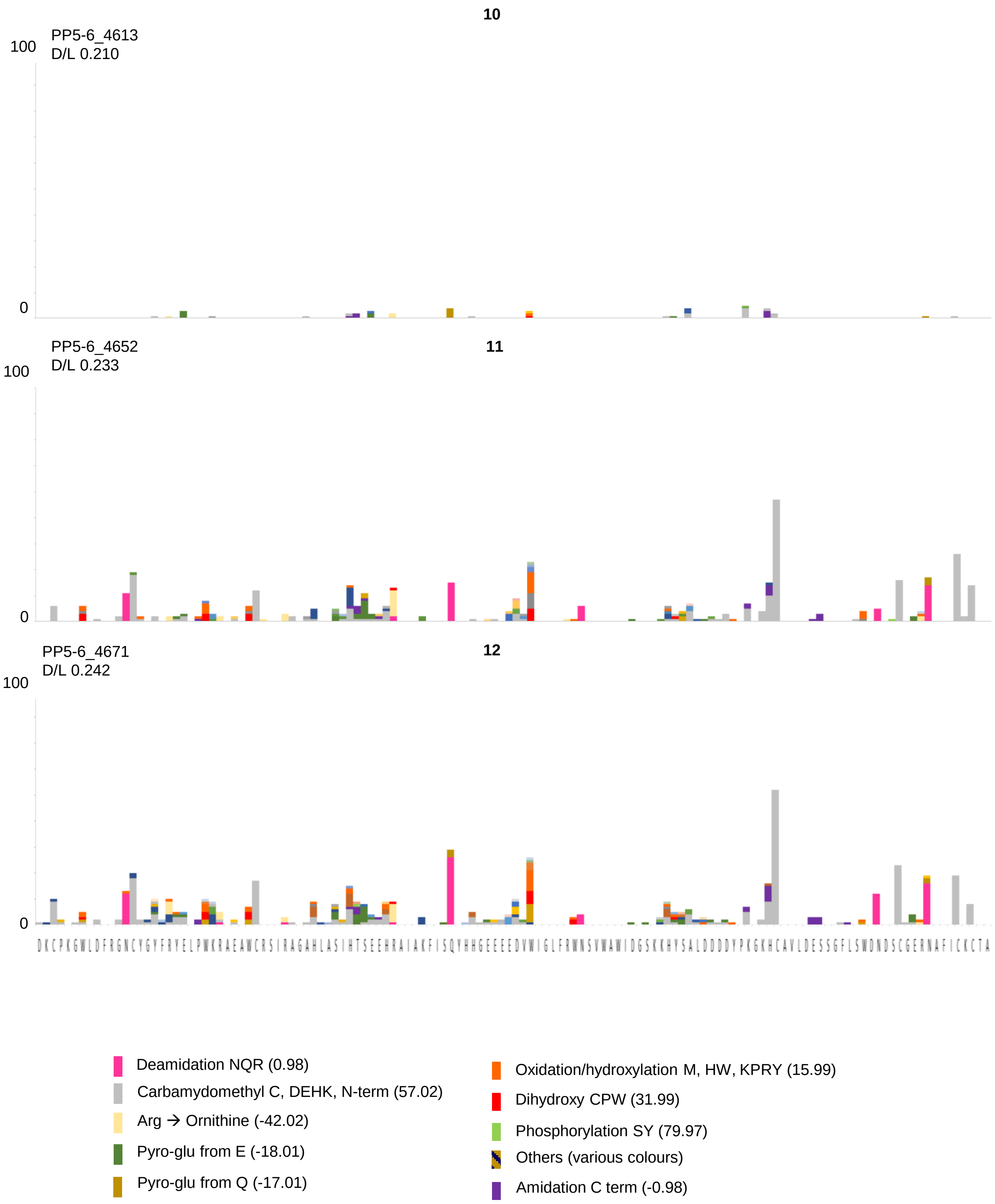
10
PP5-6_4613
D/L 0.210
100
0
11
PP5-6_4652
D/L 0.233
100
0
12
PP5-6_4671
D/L 0.242
100
0
DKCPKGWLDFRGNCYGYFRYELPWKRAEAWCRSIRAGAHLASIHTSEEHRAIAKFISQYHHGEEEEDVWIGLFRWNSVWAWIDGSKKHYSALDDDDYPKGKHCAVLDESSGFLSWDNDSCGERNAFICKCTA
Deamidation NQR (0.98)
Carbamydomethyl C, DEHK, N-term (57.02)
Arg → Ornithine (-42.02)
Pyro-glu from E (-18.01)
Pyro-glu from Q (-17.01)
Oxidation/hydroxylation M, HW, KPRY (15.99)
Dihydroxy CPW (31.99)
Phosphorylation SY (79.97)
Others (various colours)
Amidation C term (-0.98)

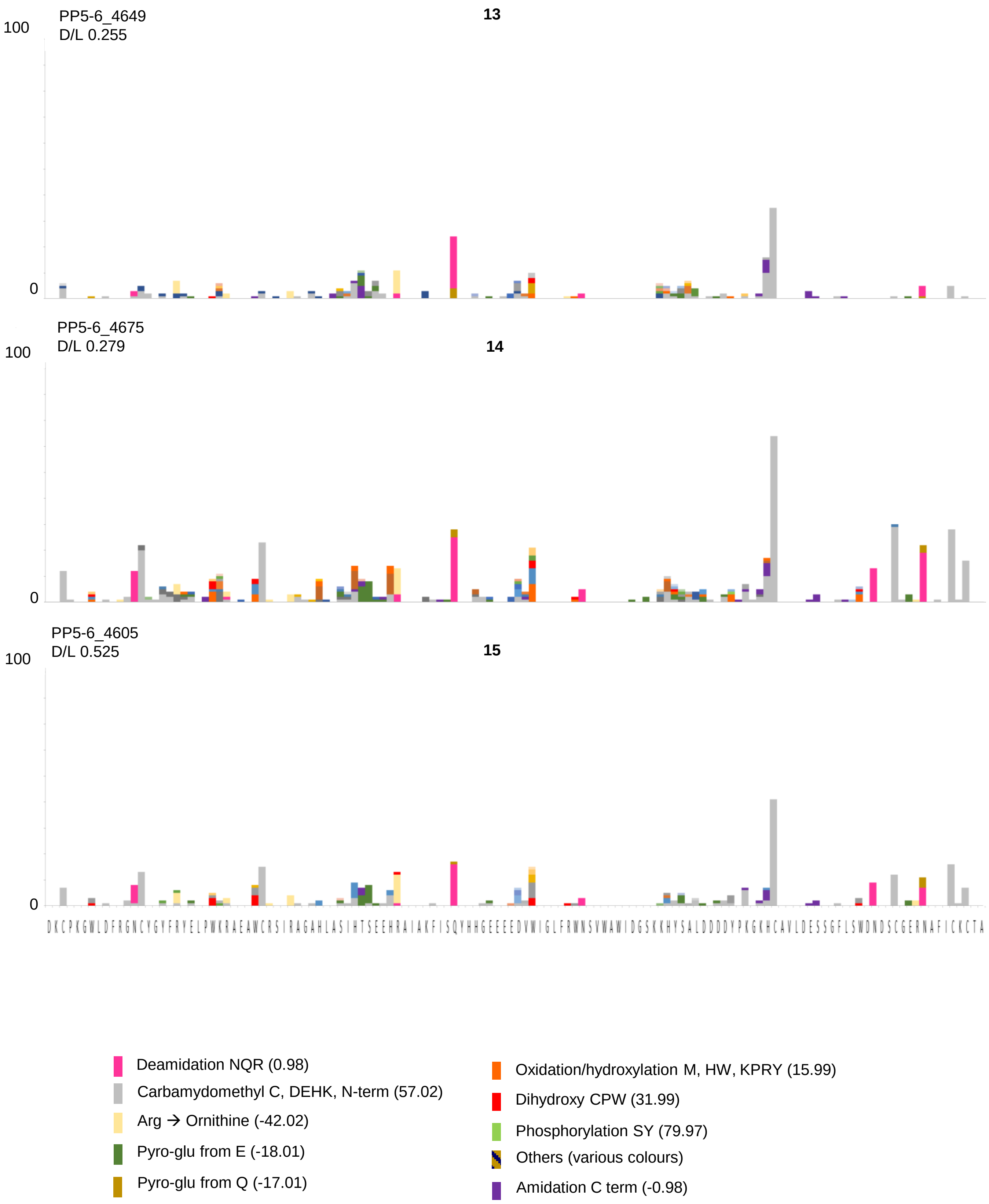
100
PP5-6_4649
D/L 0.255
13
0
100
PP5-6_4675
D/L 0.279
14
0
100
PP5-6_4605
D/L 0.525
15
0
Deamidation NQR (0.98)
Carbamydomethyl C, DEHK, N-term (57.02)
Arg → Ornithine (-42.02)
Pyro-glu from E (-18.01)
Pyro-glu from Q (-17.01)
Oxidation/hydroxylation M, HW, KPRY (15.99)
Dihydroxy CPW (31.99)
Phosphorylation SY (79.97)
Others (various colours)
Amidation C term (-0.98)

16

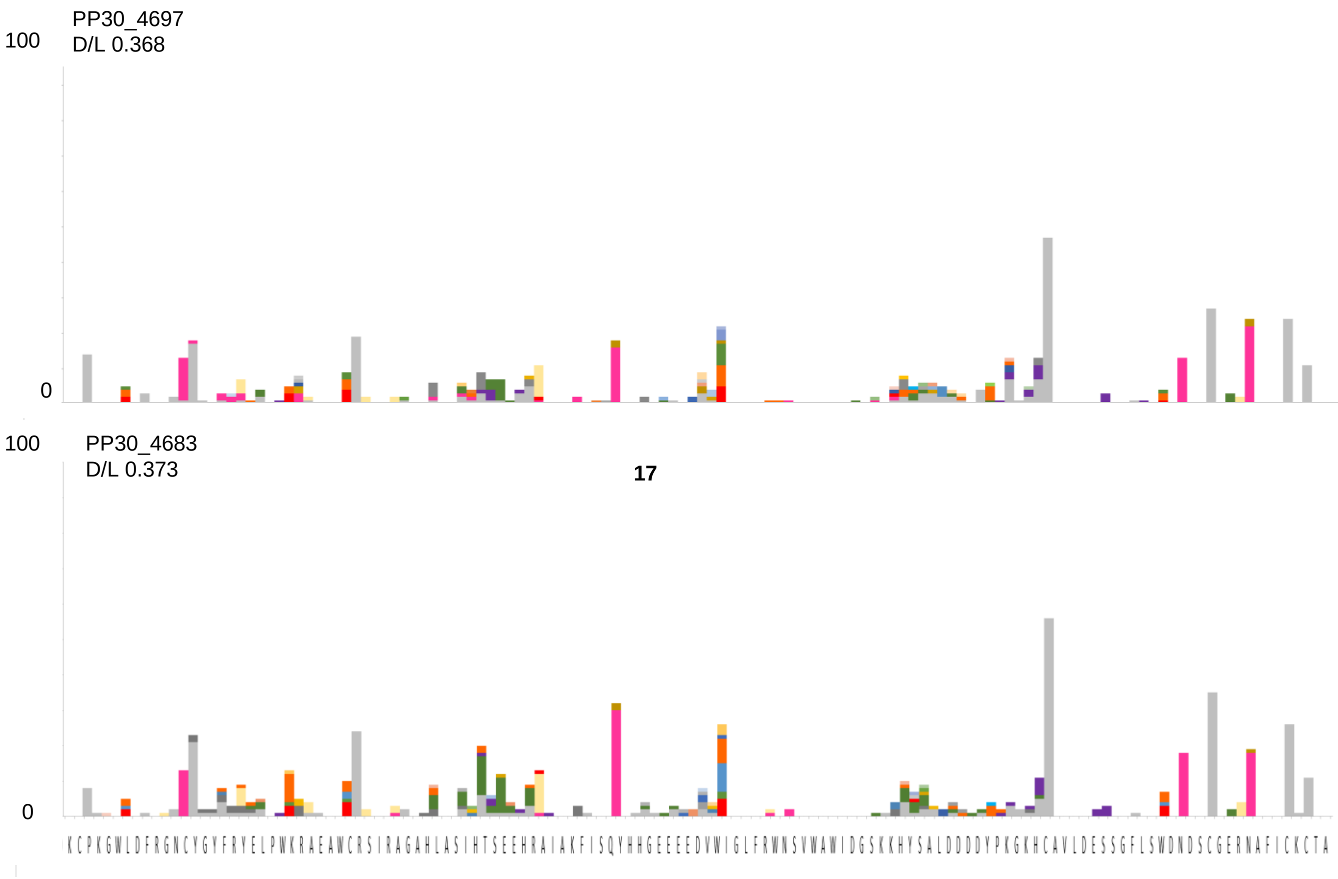

17

- Deamidation NQR (0.98)
- Carbamydomethyl C, DEHK, N-term (57.02)
- Arg → Ornithine (-42.02)
- Pyro-glu from E (-18.01)
- Pyro-glu from Q (-17.01)
- Oxidation/hydroxylation M, HW, KPRY (15.99)
- Dihydroxy CPW (31.99)
- Phosphorylation SY (79.97)
- Others (various colours)
- Amidation C term (-0.98)

18-34: Frequency of common modifications detected in each position of the sequence of SCA-2 in all bleached OES samples.

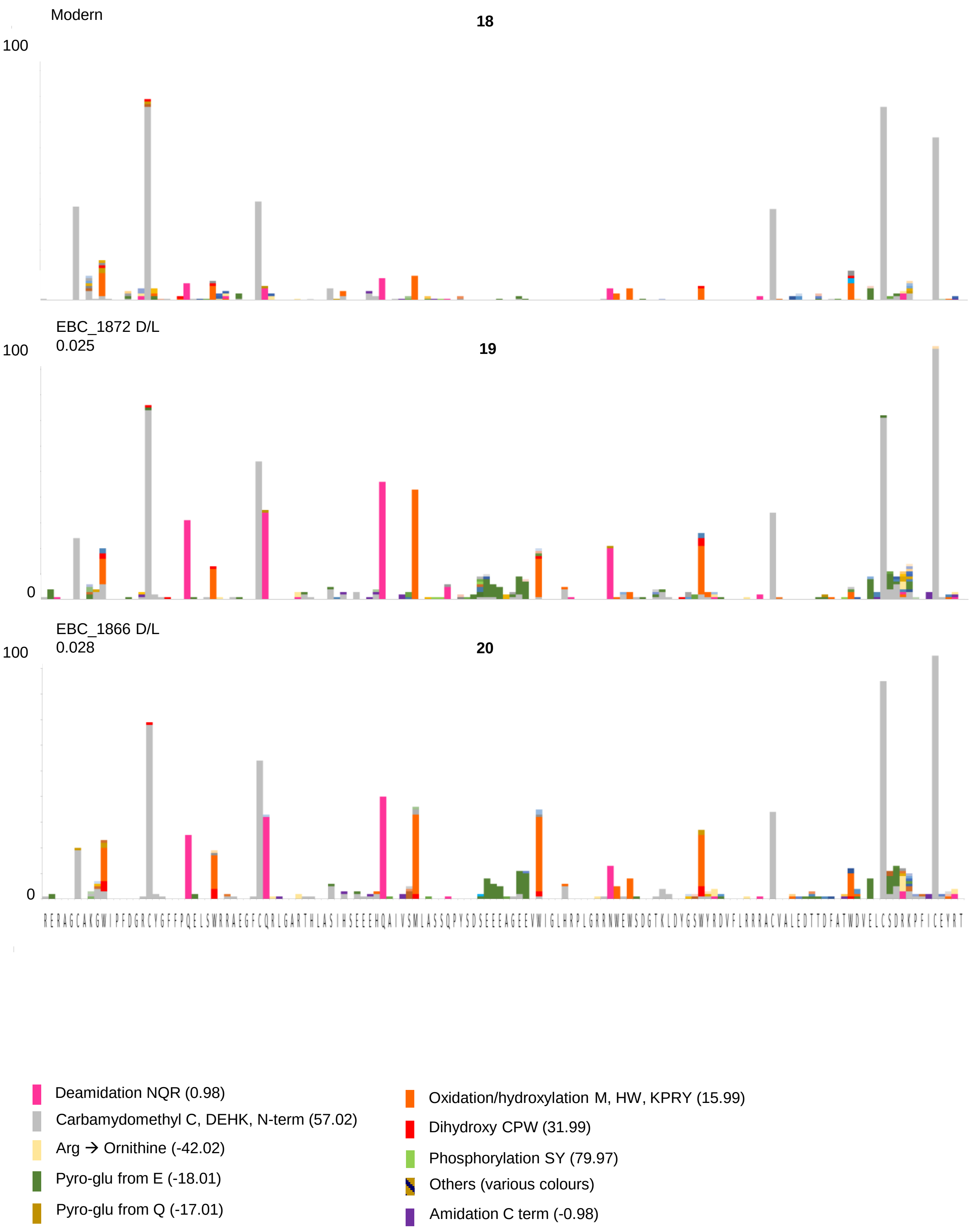

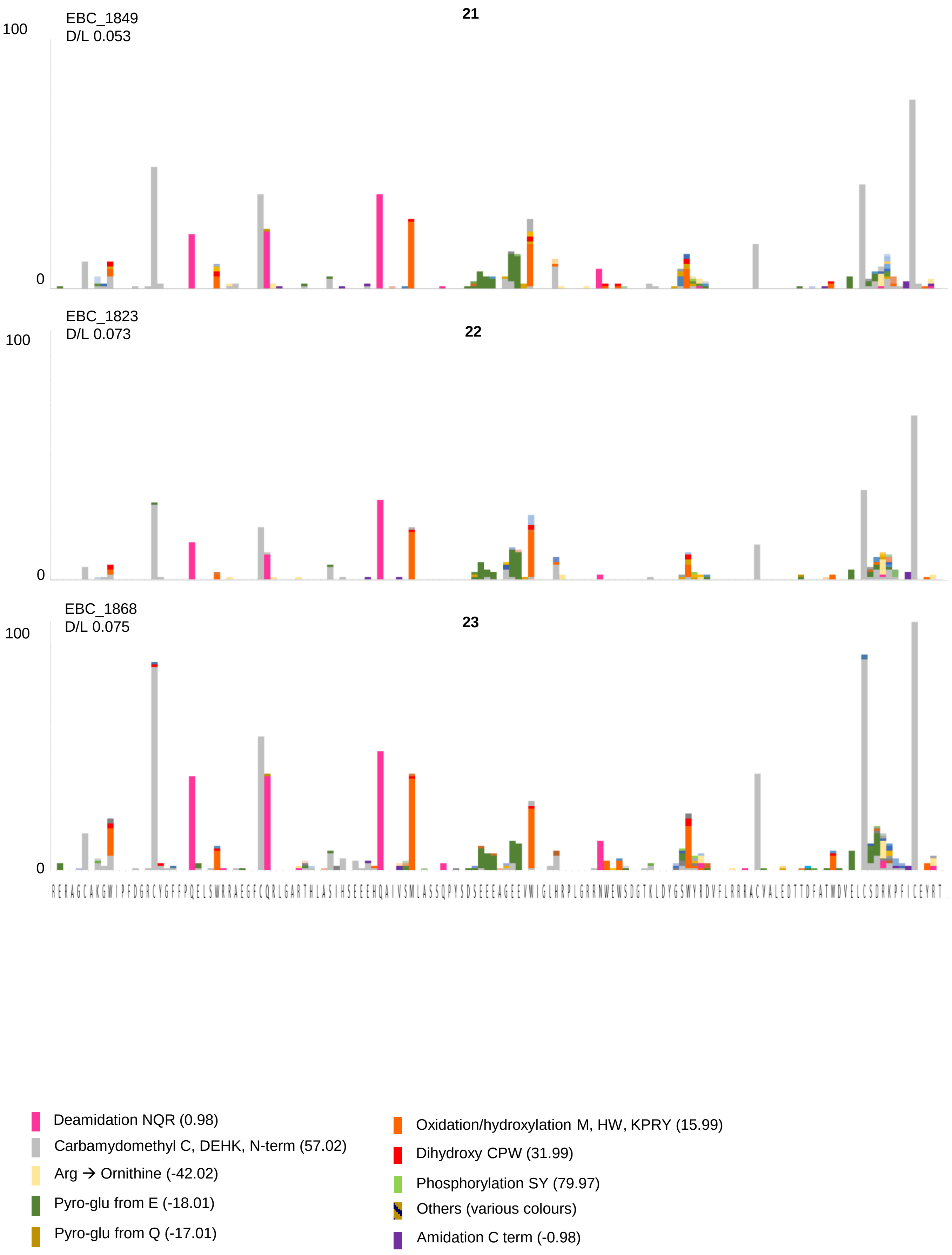

21
EBC_1849
D/L 0.053
100
0
22
EBC_1823
D/L 0.073
100
0
23
EBC_1868
D/L 0.075
100
0
RERAGCAKGWIPFDGRCYGFFPQELSWRRAEGFCQRLGARTHLASIHSEEEHQAIVSMLASSQPYSDSEEEAGEEVWIGLHRPLGRRNWEWSDGTKLDYGSWYRDVFLRRRACVALEDTTDFATWDVELCSDRKPFICEYRT
Deamidation NQR (0.98)
Carbamydomethyl C, DEHK, N-term (57.02)
Arg → Ornithine (-42.02)
Pyro-glu from E (-18.01)
Pyro-glu from Q (-17.01)
Oxidation/hydroxylation M, HW, KPRY (15.99)
Dihydroxy CPW (31.99)
Phosphorylation SY (79.97)
Others (various colours)
Amidation C term (-0.98)

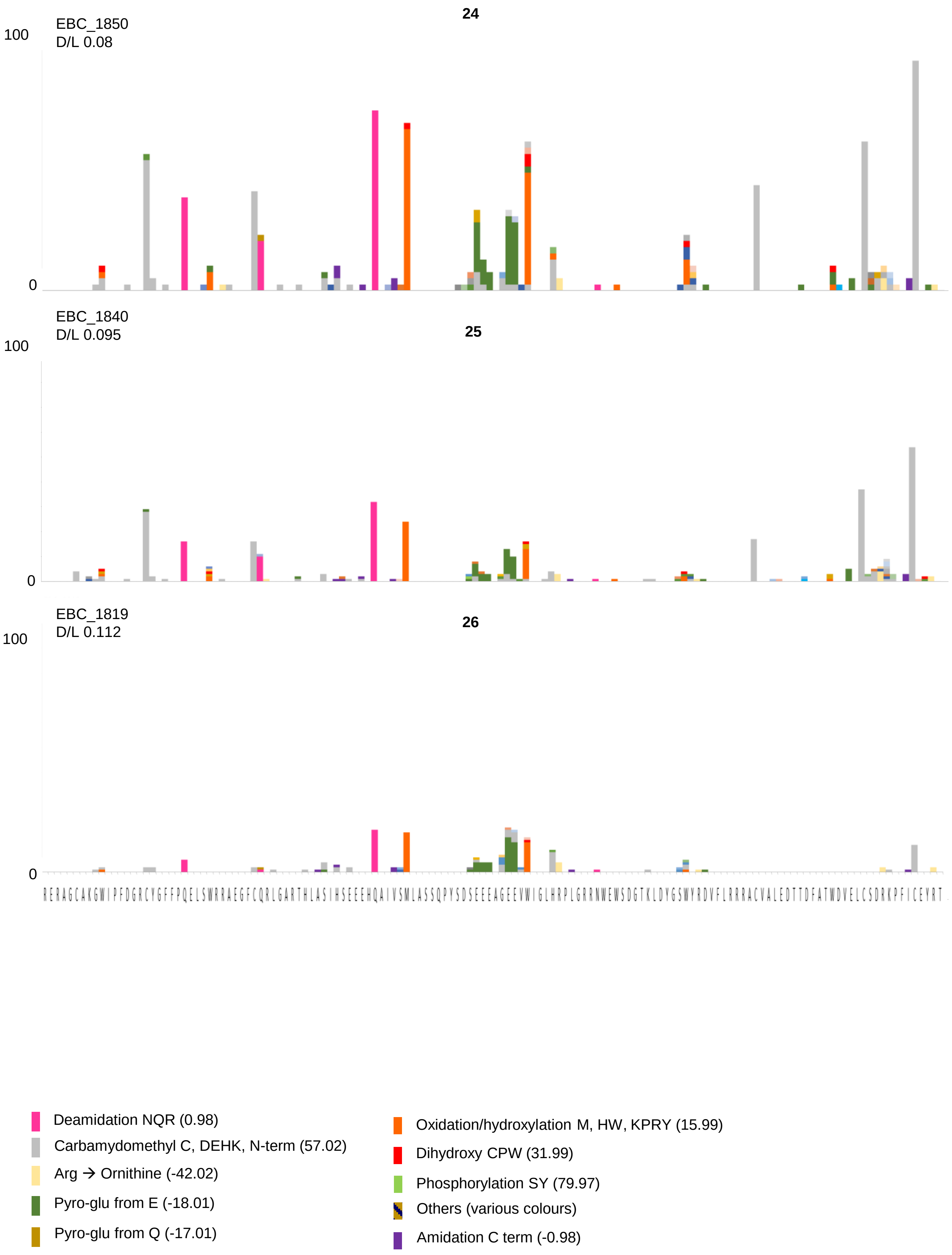
24
EBC_1850
D/L 0.08
100
0
25
EBC_1840
D/L 0.095
100
0
26
EBC_1819
D/L 0.112
100
0
RERAGCAKGWIPFDGRCYGFFPQELSWRRAEGFCQRLGARTHLASIHSEEEHQAIVSMLASSQPYSDSEEEAGEEVWIGLHRPLGRRNWEWSDGTKLDYGSWYRDVFLRRRACVALEDTTDFATWDVELCSDRKPFICEYRT
Deamidation NQR (0.98)
Carbamydomethyl C, DEHK, N-term (57.02)
Arg → Ornithine (-42.02)
Pyro-glu from E (-18.01)
Pyro-glu from Q (-17.01)
Oxidation/hydroxylation M, HW, KPRY (15.99)
Dihydroxy CPW (31.99)
Phosphorylation SY (79.97)
Others (various colours)
Amidation C term (-0.98)

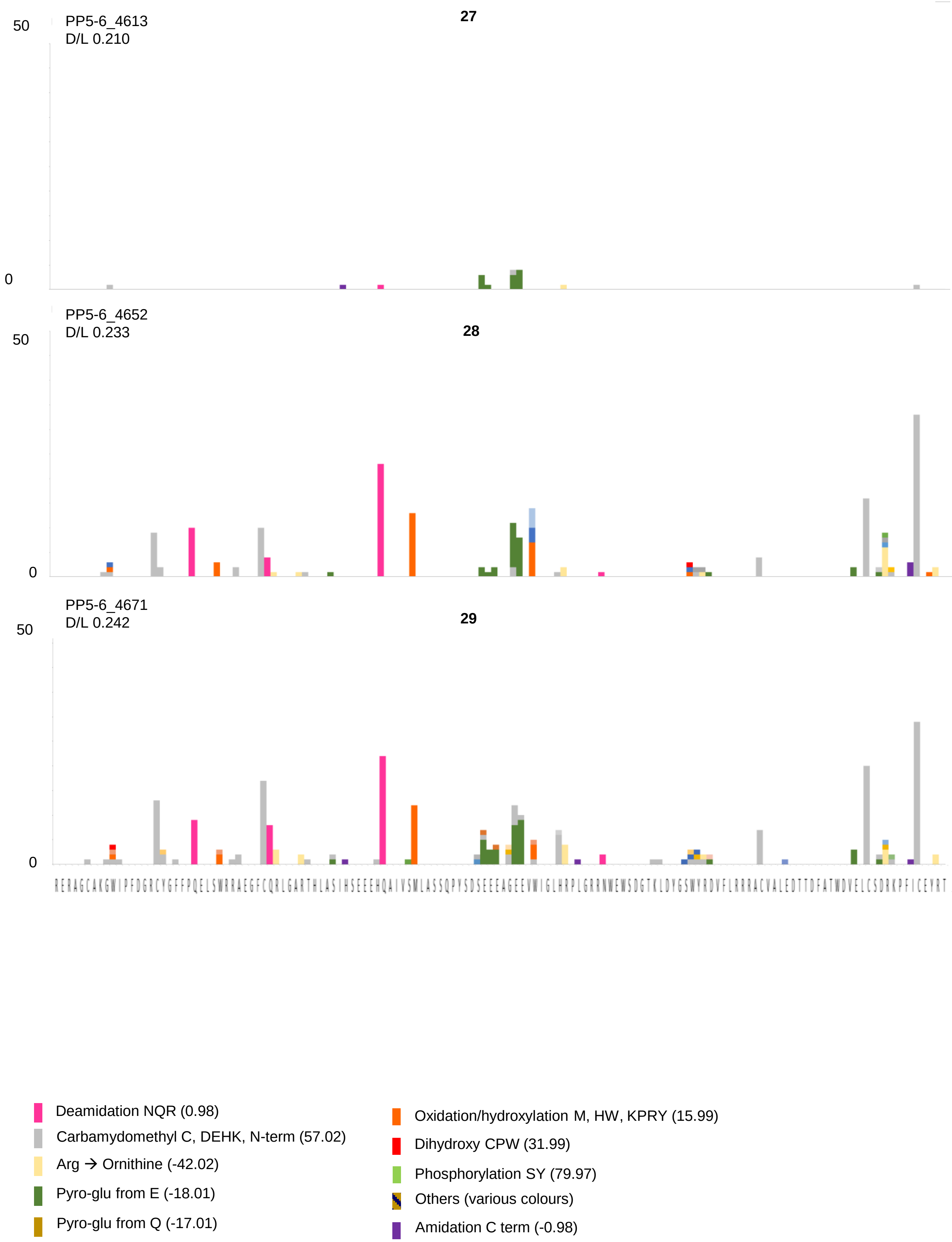
50
PP5-6_4613
D/L 0.210
27
0
PP5-6_4652
D/L 0.233
28
50
0
PP5-6_4671
D/L 0.242
29
50
0
Deamidation NQR (0.98)
Carbamydomethyl C, DEHK, N-term (57.02)
Arg → Ornithine (-42.02)
Pyro-glu from E (-18.01)
Pyro-glu from Q (-17.01)
Oxidation/hydroxylation M, HW, KPRY (15.99)
Dihydroxy CPW (31.99)
Phosphorylation SY (79.97)
Others (various colours)
Amidation C term (-0.98)

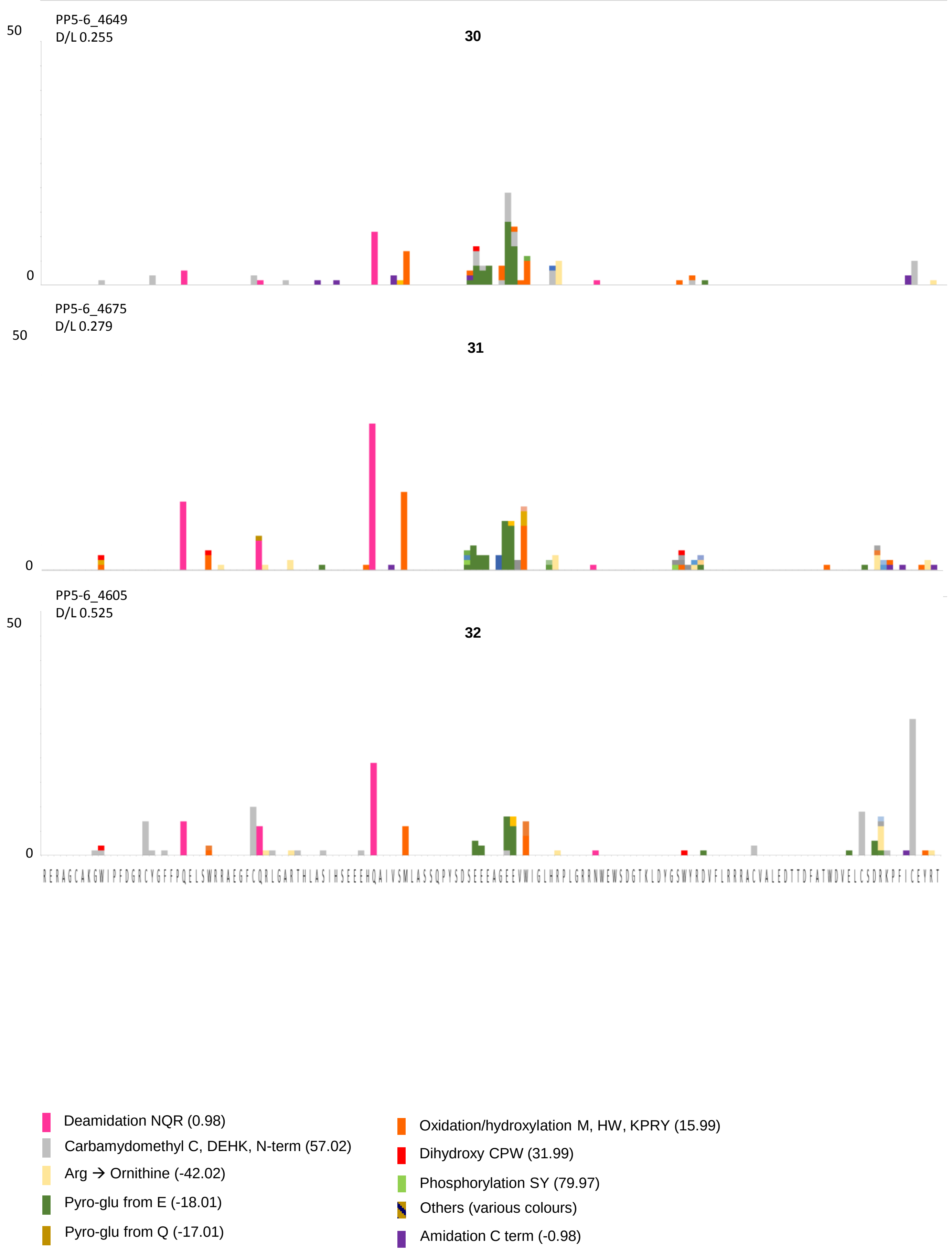
PP5-6_4649
D/L 0.255
30
50
0
PP5-6_4675
D/L 0.279
31
50
0
PP5-6_4605
D/L 0.525
32
50
0
RERAGCAKGWIPFDGRCYGFFPQELSWRRAEGFCQRLGARTHLASIHSEEEHQAIVSMLASSQPYSDSEEEAGEEVWIGLHRPLGRRNWEWSDGTKLDYGSWYRDVFLRRRACVALEDTTDFATWDVELCSDRKPFICEYRT
Deamidation NQR (0.98)
Carbamydomethyl C, DEHK, N-term (57.02)
Arg → Ornithine (-42.02)
Pyro-glu from E (-18.01)
Pyro-glu from Q (-17.01)
Oxidation/hydroxylation M, HW, KPRY (15.99)
Dihydroxy CPW (31.99)
Phosphorylation SY (79.97)
Others (various colours)
Amidation C term (-0.98)

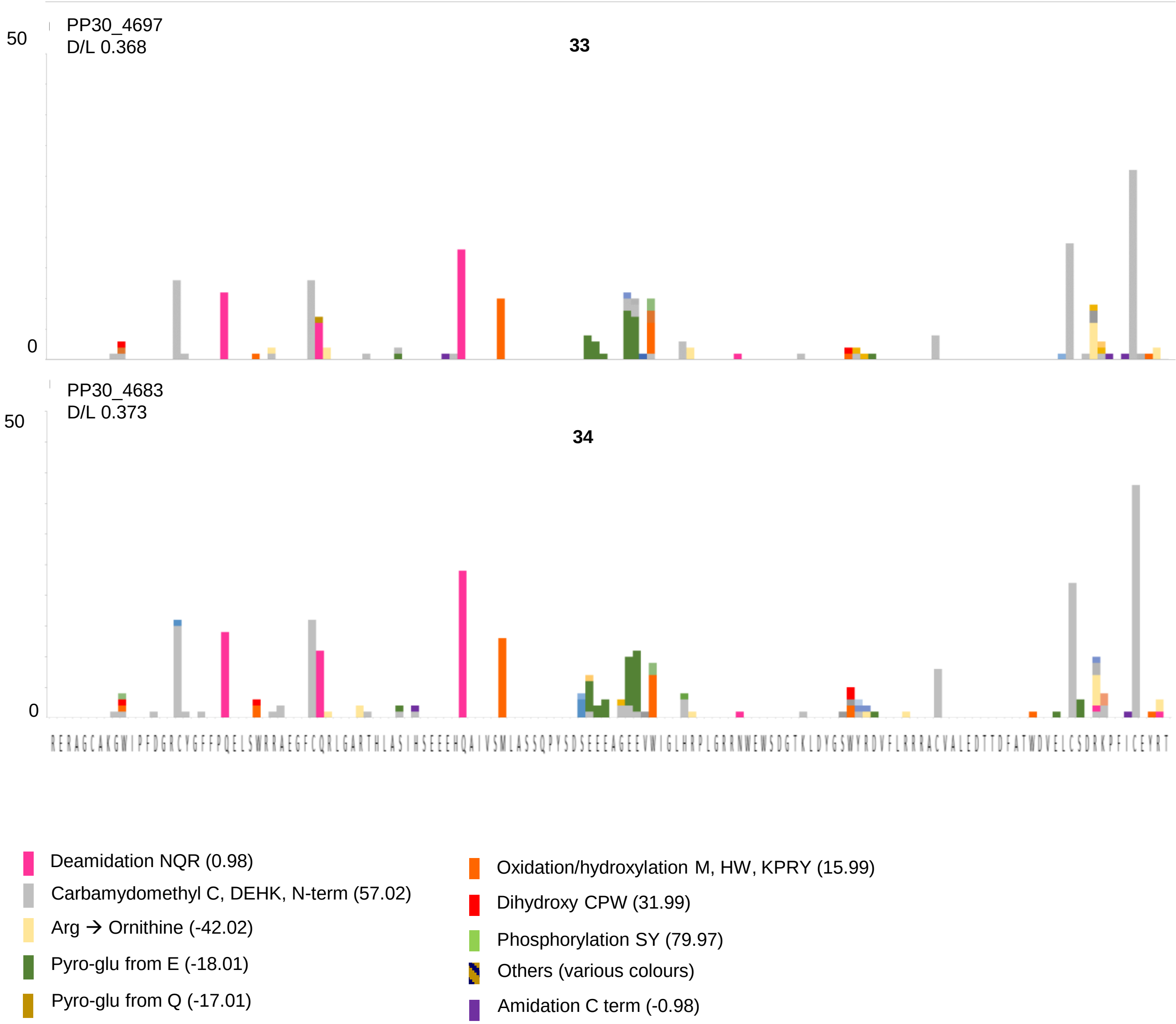
PP30_4697
D/L 0.368
33
50
0
PP30_4683
D/L 0.373
34
50
0
Deamidation NQR (0.98)
Carbamydomethyl C, DEHK, N-term (57.02)
Arg → Ornithine (-42.02)
Pyro-glu from E (-18.01)
Pyro-glu from Q (-17.01)
Oxidation/hydroxylation M, HW, KPRY (15.99)
Dihydroxy CPW (31.99)
Phosphorylation SY (79.97)
Others (various colours)
Amidation C term (-0.98)
